# A new framework for nicotinic receptor-targeted therapeutic strategies in psychiatric and neurodegenerative disorders

**DOI:** 10.3389/fncel.2026.1768143

**Published:** 2026-06-08

**Authors:** Khalid Oudaha, Frédéric Causeret, Fani Koukouli

**Affiliations:** 1 Université Paris Cité, Institute of Psychiatry and Neuroscience of Paris (IPNP), INSERM U1266, Team Cholinergic Modulation of Cortical Inhibitory Circuits in Health and Disease, Paris, France; 2 Université Paris Cité, Institute of Psychiatry and Neuroscience of Paris (IPNP), INSERM U1266, Team Genetics and Development of the Cerebral Cortex, Paris, France; 3 Université Paris Cité, Imagine Institute, Team Genetics and Development of the Cerebral Cortex, Paris, France; 4 GHU-Paris Psychiatrie et Neurosciences, Hôpital Sainte Anne, Paris, France

**Keywords:** Alzheimer’s disease, cognition, neuroprotection, nicotine, nicotinic acetylcholine receptors, precision medicine, prefrontal cortex, schizophrenia

## Abstract

The nicotinic acetylcholine receptors (nAChRs) are key modulators of synaptic transmission and cognitive processing within the central nervous system. These ligand-gated channels, composed of various α and β subunits, mediate a plethora of neuronal functions including attention, memory and executive control. The current perspective article synthesizes recent advances on the contribution of pivotal nAChRs subtypes particularly α4β2, α7, and α5-containing receptors to cortical circuit function, highlighting their relevance in health and disease. In healthy brain, nAChRs regulate excitatory-inhibitory balance and enhance cognitive mechanisms in the prefrontal cortex (PFC) and hippocampus. Recent findings demonstrate that α5-containing receptors exhibit selective resistance to amyloid-β induced dysfunction, suggesting a neuroprotective role in Alzheimer’s disease (AD). Integrating molecular, cellular, and behavioral evidence, we argue that receptor-subtype-specific modulation of distinct nAChRs subunits represents a promising therapeutic avenue for restoring network balance and cognitive function across neuropsychiatric and neurodegenerative disorders. We further discuss the role of nicotine in brain circuits and suggest that future research should prioritize precision pharmacology and genetic profiling to identify optimal therapeutic windows and mitigate the long-term consequences of nicotine exposure on developing neural circuits.

## Introduction

Nicotinic acetylcholine receptors (nAChRs) are ionotropic receptors that mediate fast synaptic transmission in both the central and peripheral nervous systems. At neuromuscular junctions they drive muscle contraction, while in the brain they influence cognition, aging, and play a key role in several neurological disorders (Dineley et al., 2015; Koukouli and Maskos, 2015).

Structurally, nAChRs are pentameric cation-permeable channels formed from α (α2–α7, α9–α10) and β (β2–β4) subunits (Taly et al., 2009). The main CNS subtypes are high-affinity heteromeric α4β2 receptors and low-affinity, high Ca^2+^-permeable homomeric α7 receptors. In α4β2 receptors, four subunits form the ligand-binding sites, while a fifth accessory subunit can modulate receptor properties. Addition of the α5 accessory subunit, enhances Ca^2+^ permeability and acetylcholine sensitivity but speeds desensitization (Maskos, 2020).

In this perspective, we will discuss recent findings on the functional role of nAChRs in the healthy and diseased brain. We emphasize the growing need to pivot toward precision pharmacology and personalized therapeutic strategies. Such an approach may be essential for effectively addressing complex psychiatric and neurodegenerative disorders. By contrast, muscarinic acetylcholine receptors (mAChRs), which are metabotropic, have recently been reviewed (Tobin, 2024) and will not be addressed further here.

### Nicotinic acetylcholine receptors in cognitive processing and prefrontal cortex inhibitory circuits: insights from studies using knockout animals

Genetic knockout studies have been central to clarifying the role of individual nAChR subunits. For example, α5 knockout mice display impaired performance in the five-choice serial reaction time test, which measures visual attention. While performance under low attentional demand was unaffected, α5 knockouts showed reduced accuracy when attentional demand increased (Bailey et al., 2010). These findings extend earlier evidence that acetylcholine modulates attentional gating in the prefrontal cortex (PFC; Dalley et al., 2004; Parikh et al., 2007) and highlight a specific role for the α5 subunit.

Similarly, β2 knockout mice exhibit attentional deficits in the same task, which can be rescued by selective re-expression of β2 subunits in the prelimbic cortex (Guillem et al., 2011). The β2 knockouts also show altered social behavior, with enhanced social contact compared to wild-type mice. This phenotype is normalized by viral re-expression of β2 in the PFC (Avale et al., 2011). Homomeric α7 nAChRs also play key roles in cognition. However, in terms of attention, α7 knockout mice performed poorly in the five-choice reaction time test, showing slower learning and reduced accuracy, results which are consistent across multiple studies (Hoyle et al., 2006; Young et al., 2004).

Electrophysiological and imaging studies further suggest that β2-containing receptors contribute to ultraslow cortical fluctuations (<0.1 Hz) in the PFC, which are important for cognitive processing during quiet wakefulness (Koukouli et al., 2016b). Further studies should follow to identify the role of nAChRs in neuronal synchronicity and neuronal ensembles during cognitive behaviors.

**FIGURE 1:**
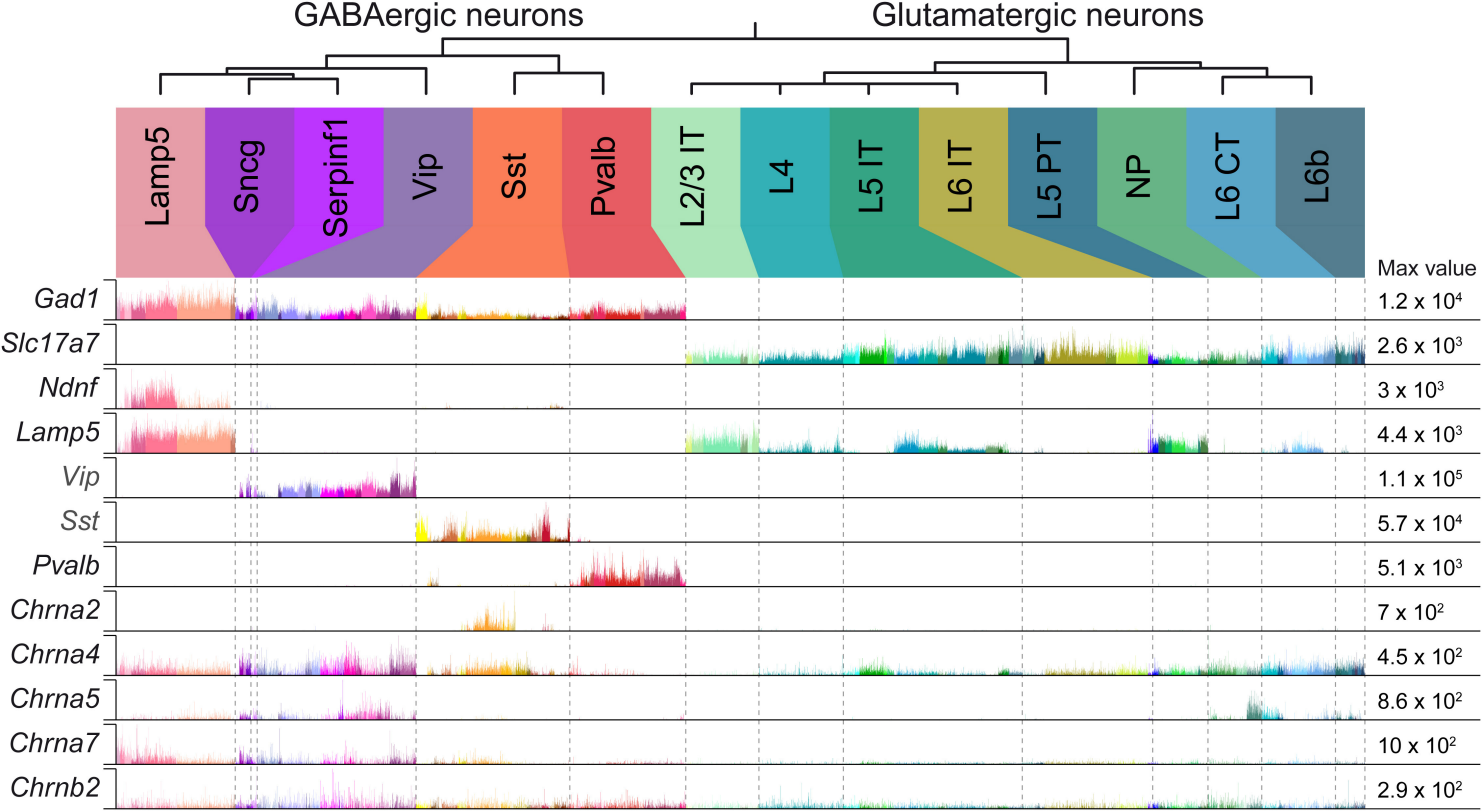
Expression of selected nicotinic acetylcholine receptor genes in the adult mouse motor and visual cortex. Bar plot representing the expression level of a subset of nicotinic acetylcholine receptor genes α2 (*Chrna2*), α4 (*Chrna4*), α5 (*Chrna5*), α7 (*Chrna7*) and β2 (*Chrnb2*) in the main subclasses of GABAergic and Glutamatergic neurons of the adult mouse motor and visual cortex (Tasic et al., 2018). Population-specific marker genes distinguishing cell classes and subclasses are also shown. Data were extracted from the Allen Brain Atlas (https://celltypes.brain-map.org/rnaseq/mouse/v1-alm). Each bar represents a single cell (only “core” cells were considered) and is color-coded according to clusters from the original publication. The scale is linear and maximum values in counts per million (CPM) are indicated. IT, intratelencephalic; PT, pyramidal tract; NP, near-projecting; CT, corticothalamic.

In the visual and motor cortex, nAChRs are highly expressed by GABAergic interneurons (Figure 1). Importantly, electrophysiology and imaging studies in the PFC, have highlighted that nAChR expression in this area is both cell-type and layer specific (Koukouli et al., 2017, 2026; Poorthuis et al., 2013, 2018; Abbondanza et al., 2024). In the superficial layers of the PFC, somatostatin (SST) interneurons express the α7- and β2-containing receptors, parvalbumin (PV) interneurons express α7 nAChRs, whereas α5-containing receptors are expressed in vasoactive intestinal polypeptide (VIP) interneurons. VIP interneurons, regulate inhibition of SST and PV interneurons, however, recently it has been demonstrated connectivity between specific subtypes of VIP interneurons and pyramidal neurons in the cortex (Pi et al., 2013; Zhu et al., 2025; Shlomo et al., 2025). Importantly, *in vivo* two-photon calcium imaging of α5 knockout mice revealed reduced activity in pyramidal neurons of layers II/III in the prelimbic cortex (Koukouli et al., 2017; Rooy et al., 2021). Since α5 is expressed specifically in VIP interneurons in layers II/III, this phenotype was attributed to disinhibition via SST interneurons. Re-expression of α5 nAChRs selectively in GABAergic interneurons in layers II/III of the prelimbic cortex restores pyramidal neuron activity, confirming a dominant role for α5-mediated disinhibition in regulating cortical circuit dynamics. In addition, α5 nAChRs are also expressed in deeper layer pyramidal neurons in the mPFC and in excitatory corticothalamic neurons in layer VI, playing crucial role in sensory gating and attentional mechanisms (Venkatesan et al., 2023; Qi et al., 2024).

By simultaneously probing circuit computations and behavioral outcomes, future research can move toward a mechanistic understanding of how specific nicotinic receptor subtypes shape cognition. This integrative approach will make it possible to link directly the molecular identity of receptor subunits, their cell-type–specific expression, and their role in local microcircuit dynamics with behavioral performance in well-defined cognitive tasks. Such knowledge will help to uncover the computational rules by which α 5-, β 2-, and α7-containing receptors influence attentional control, memory encoding, and cognitive flexibility.

### Nicotinic acetylcholine receptors in memory

Cholinergic pathways, acting through nAChRS, play an active role in memory modulation, and their dysfunction is linked to cognitive decline across several neurological disorders. Memory itself is defined as the storage of environmental information acquired during learning and is classically divided into stages: acquisition, consolidation, retrieval, extinction, and reconsolidation (Kandel, 2013; Pastor and Medina, 2024). The α7 and α4β2 nAChR subtypes receptors have been extensively studied due to their abundance in memory related circuits including hippocampus, and their distinct biophysical and pharmacological features : α4β2 receptors characterized by high nicotine affinity and sustained signaling, whereas α7 display rapid activation and fast desensitization kinetics (Albuquerque et al., 2009; Echeverria et al., 2023; Pastor and Medina, 2024; Taly et al., 2009). We propose here that these receptor subtypes function as circuit-level regulators of memory processing, controlling synaptic plasticity, network excitability, and information flow. This framework implies that memory emerges from subtype-specific modulation of circuit dynamics.

The α7 nAChR, composed entirely of α7 subunits, is notable for its exceptionally high permeability to calcium ions. This property places it at the center of mechanisms governing synaptic plasticity and cognitive processes such as learning and memory (Koukouli and Changeux, 2020; Pastor and Medina, 2024). In the hippocampus, activation of α7 receptors strengthens synaptic efficacy by promoting long-term potentiation (LTP) in excitatory pyramidal neurons (Townsend et al., 2016), whereas in inhibitory GABAergic interneurons, the same activation favors long-term depression (LTD; Ji et al., 2001) suggesting that α7 receptors operate as plasticity gatekeepers. The coexistence of these complementary forms of plasticity is considered essential for the proper encoding and stabilization of memory. In the prelimbic region of the PFC, α7 receptor stimulation exerts bidirectional control over network excitability and synaptic remodeling, simultaneously influencing excitatory and inhibitory transmission (Levin et al., 2006; Udakis et al., 2016). Despite extensive research, findings remain partly contradictory: while *in vivo* studies indicate that α7 receptor activation facilitates hippocampal–prefrontal LTP (Stoiljkovic et al., 2016), experiments in hippocampal slices have shown an opposite effect, with α7 stimulation dampening LTP induction (Nakauchi and Sumikawa, 2014). However, despite these inconsistencies, the results reflect context-dependent circuit regulation and support the idea that α7 signaling intervenes at the level of network state and circuit dynamics.

Behaviorally, α7 receptors in the PFC have been linked to aversive associative memory. Infusion of the α7 specific antagonist methyllycaconitine (MLA) reduced contextual fear responses in trace fear conditioning (Raybuck and Gould, 2010). However, other studies found no effect of MLA on trace fear retrieval (Miguelez Fernández et al., 2021), suggesting that discrepancies may arise from differences in rodent models or blocker’s concentrations. The α7 nAChRs also contribute to rewards-related associative memory. Using conditioned place preference, pharmacological studies demonstrated their role in cocaine-associated memory acquisition and retrieval, but not in consolidation (Pastor et al., 2021). Thus, these data support a model in which α7 receptors regulate memory plasticity and adaptive circuit flexibility rather than memory storage.

Beyond aversive and rewards learning, prefrontal nAChRs also participate in episodic memory. In novel object recognition tasks, stimulation of α7 and α4β2 receptors enhanced acquisition, an effect blocked by selective antagonists (Dere et al., 2007; Esaki et al., 2021; Izumi et al., 2025). Consistently, pharmacological blockade of hippocampal α7 and α4β2 receptors impaired working (Nott and Levin, 2006), aversive (Raybuck and Gould, 2010), and rewards-related memory (Wright et al., 2019).

The amygdala, another hub of associative learning, also expresses high levels of α7 and α4β2 nAChRs (Mendez et al., 2013; Pastor and Medina, 2024; Pidoplichko et al., 2013). In rodents, in the basolateral amygdala selective inhibition of these receptors with MLA and dihydro-β-erythroidine (DHβE), respectively, disrupted working memory (Addy et al., 2003), while α7 inhibition in the central amygdaloid nucleus enhanced aversive behaviors (Ishida et al., 2011). These findings support an extended circuit-level role for nAChRs in linking emotional and cognitive memory networks.

Importantly, the pro-cognitive effects of nicotinic agonists in both preclinical and clinical studies highlight the therapeutic potential of targeting nAChRs in disorders characterized by cholinergic dysfunction, such as Alzheimer’s disease and schizophrenia (Bruszt et al., 2025; Freedman et al., 1997, 2008; Izumi et al., 2025; Koukouli and Changeux, 2020). From a translational perspective, these data support a shift from non-selective cholinergic stimulation toward subtype-specific modulation of nAChRs within defined circuit contexts. We propose that therapeutic strategies should aim to restore physiological plasticity thresholds, excitation–inhibition balance, and network stability, rather than simply enhance cholinergic tone. Within this framework, nAChRs emerge not as generic cognitive enhancers but as circuit control nodes capable of restoring memory function through precise modulation.

### Nicotinic acetylcholine receptors in Alzheimer’s disease: a “nicotinic” mechanism of Aβ-dependent pathology

Alzheimer’s disease (AD) is a progressive neurodegenerative disorder and the most common form of dementia. It is characterized by extensive neuronal loss across several brain regions, especially those implicated in memory and cognition. A defining molecular feature of AD is the accumulation of misfolded proteins, most notably the amyloid- β (Aβ) peptide (Hardy and Allsop, 1991; Mattson, 2004). Prior to the onset of dementia, one of the earliest neuropathological events in AD is the degeneration of cholinergic neurons in the basal forebrain, which contributes significantly to cognitive decline and disease progression (Bartus, 2000; Mesulam, 2004; Ballinger et al., 2016; Shafiee et al., 2024). Although Aβ accumulation and cholinergic system dysfunction are two of the most replicated pathological hallmarks, the causal relationships between these phenomena are poorly understood. New evidence increasingly supports a more nuanced view, wherein cholinergic dysfunction is not merely a downstream effect of neurodegeneration but may be an early and contributing factor in pathological circuit remodeling (Lykhmus et al., 2024; Sabec et al., 2025; Koukouli et al., 2026).

Cholinergic dysfunction has been shown to correlate more closely with the severity of cognitive impairment in AD than the extent of amyloid deposition (Mufson et al., 2008; Turnbull et al., 2018). Postmortem analyses have consistently revealed reduced choline acetyltransferase (ChAT) activity in the hippocampus, amygdala, and cortex, regions critical for learning, emotional regulation, and higher cognition, alongside marked decreases in acetylcholinesterase (AChE) catalytic activity in the same areas (Davies and Maloney, 1976). These biochemical alterations are associated with the degeneration of cholinergic projections originating from the nucleus basalis of Meynert (Whitehouse et al., 1981).

Therapeutic strategies targeting this cholinergic deficit have focused on inhibiting AChE to prolong synaptic acetylcholine availability. Drugs approved by the U.S. Food and Drug Administration (FDA) including tacrine, donepezil, rivastigmine, and galantamine, have demonstrated modest yet sustained improvements in cognitive performance. However, these agents provide only symptomatic relief and are frequently associated with adverse side effects. Among these, galantamine uniquely appears to lower the risk of progression to severe dementia (Bartus, 2000; Xu et al., 2021).

Within the PFC, acetylcholine is crucial for maintaining the balance between excitation and inhibition (E/I balance), which is essential for stable cognitive states. Disruption of this balance contributes to the cognitive impairment’s characteristic of AD (Lauterborn et al., 2021; Styr and Slutsky, 2018; Vico Varela et al., 2019). Postmortem studies further reveal a decline in nAChR density across multiple brain areas, including the frontal cortex (Court et al., 2001; Nordberg and Winblad, 1986).

Among nAChR subtypes, the high-affinity α4β2 receptors are particularly abundant in the cortex and hippocampus, regions integral to attention, memory, and executive function (Gotti et al., 2006; Patel et al., 2025). Imaging and immunohistochemical studies have demonstrated a 30%–50% reduction in α4β2 receptor expression in AD brains (Court et al., 2001; Schmaljohann et al., 2004; Wu et al., 2010), suggesting that receptor loss serves as a robust marker of disease progression. Functionally, α4β2 receptors facilitate dopamine and glutamate release, and their downregulation is believed to underlie deficits in working memory and attentional processes (Garduño et al., 2012; Grupe et al., 2013; Palombo et al., 2024).

The α7 nAChRs play a complex role in AD pathology. It binds with high affinity the Aβ1-42 peptide, which is a 42-amino acid peptide generated by the proteolytic cleavage of the amyloid precursor protein (APP) highly associated with AD (Wang et al., 2000). The results of this study showed that Aβ1-42 has a high affinity specifically for α7 nAChRs, which was found to be 5000-fold lower for α4β2 nAChRs and showed only non-specific binding for muscarinic receptors. Excessive interaction between Aβ1-42 and α7 receptors leads to receptor desensitization, calcium dysregulation, and synaptic failure involving both neurons and glial cells (Ikonomovic et al., 2009; Liu et al., 2001; Rennie, 2025; Wang et al., 2000). At the circuit level, this interaction contributes to E/I imbalance and weakened cortical synchrony (Koukouli et al., 2016a,^2026^).

Conversely, under physiological conditions, α7 nAChR activation exerts neuroprotective and anti-inflammatory actions by engaging intracellular signaling cascades such as JAK2/STAT3 and PI3K/Akt, thereby supporting neuronal survival (Ma and Qian, 2019; Rennie, 2025; Shimohama and Kawamata, 2018). Pharmacological activation of α7 receptors through selective agonists, such as PNU-282987, encenicline, and BMS-933043 or positive allosteric modulators (PAMs) has improved cognitive outcomes in preclinical models, although clinical trials have yielded inconsistent results (Burns et al., 2023; Magnussen, 2025; Wang et al., 2020). CHRFAM7A, a uniquely human fusion gene, is a negative regulator of α7 nAChR and was unaccounted in preclinical models (Szigeti et al., 2020; Görgülü et al., 2024). Specifically, the CHRFAM7A gene, the human-specific duplication of the α7-encoding CHRNA7, encodes for a truncated α7 that lacks part of the N-terminal (Jiang et al., 2019). CHRFAM7A can co-assemble with regular α7 subunits to form functional receptors that negatively regulate the function of α7 nAChRs (Lasala et al., 2018). Interestingly, CHRFAM7A is carried by up to 75% of the human population, meaning that the efficacy of AD medications targeting α7 nAChRs could vary from patient to patient depending on if they are expressing the mutated gene or not (Szigeti et al., 2020). It is suggested that before treatment with cholinergic medication, patients should be screened to assess if they are carriers of the CHRFAM7A (Szigeti et al., 2020). Lastly, the remaining 25% of humans that do not express CHRFAM7A could have a better response to the currently formulated CHRNA7-specific treatments. Moreover, the CHRFAM7A gene exhibits copy number variations, with individuals carrying either one copy or none, and deletions involving CHRNA7 and CHRFAM7A are strongly associated with schizophrenia (Stefansson et al., 2008).

Recent studies have highlighted the significance of the auxiliary α5 subunit, encoded by the *CHRNA5* gene. Although α5 alone cannot form functional receptors, it assembles with α4 and β2 subunits to generate α4α5β2 nAChRs. Incorporation of the α5 subunit alters the receptor’s properties by increasing calcium permeability (Sciaccaluga et al., 2015), modifying activation kinetics (Venkatesan et al., 2023; Venkatesan and Lambe, 2020), and influencing downstream signaling cascades (Gerzanich et al., 1998; Scholze and Huck, 2020). Interestingly, the Aβ peptide appears to inhibit α4α5β2 receptors less effectively than other nicotinic receptor subtypes (Lamb et al., 2005; Koukouli et al., 2026), implying a potential protective function in AD (Rybnicek et al., 2024). Importantly, recent genetic analyses of human tissue have linked *CHRNA5* polymorphisms with increased cortical Aβ burden, and *CHRNA5* expression has been found enriched in chandelier cells critical for maintaining E/I balance, further supporting a neuroprotective role of the α5 subunit in AD (Rybnicek et al., 2024).

**FIGURE 2:**
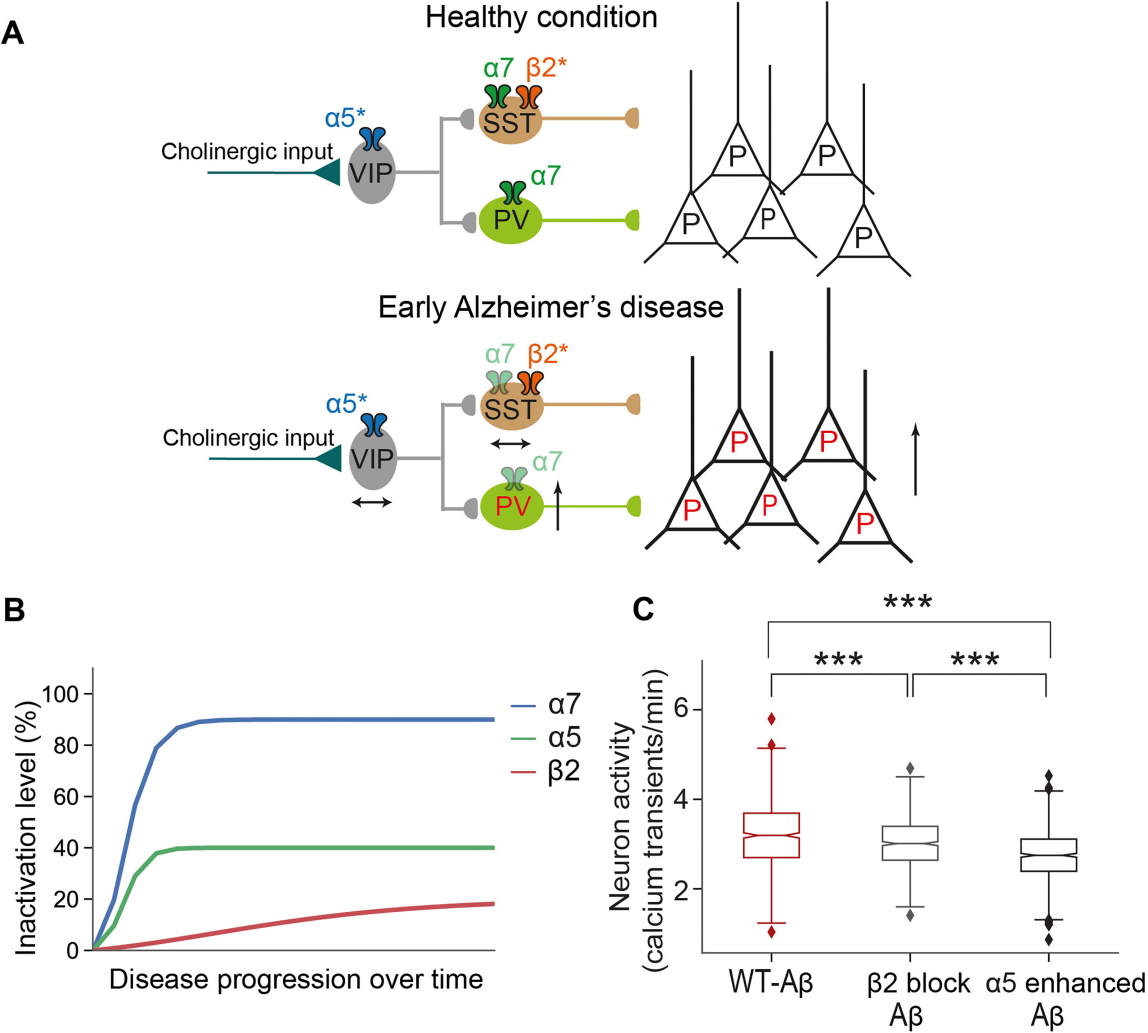
Human Aβ oligomers differentially inactivate distinct nAChRs during Alzheimer’s disease progression in the prefrontal cortex. **(A)** Schematic illustrating how in early Alzheimer’s disease the activity of pyramidal neurons and GABAergic neurons is affected. Specifically, inactivation of α7 nAChRs by the Aβ leads to increased pyramidal neuron and PV-expressing interneuron activity in layers 2/3 of the prelimbic cortex. * Indicates α5 or β2-containing nAChRs. **(B)** α7 nAChRs are almost fully inactivated in early AD, whereas α5 and β2-containing nAChRs are partially inactivated and available as targets for therapeutic strategies. **(C)** Pharmacological boosting of α5 or blocking of β2 nAChRs can help restore AD related neuronal hyperactivity to normal levels, as predicted by computational modeling and verified experimentally using galantamine, which in low doses acts as a positive allosteric modulator of α5 nAChRs. Adapted by Koukouli et al. (2026). ©The Authors, under exclusive licence to Springer Nature Limited 2025.

We have recently demonstrated that α7 nAChRs are key targets of the soluble form of Aβ peptide in the early stages of amyloid pathology (Koukouli et al., 2026). Using *in vivo* two-photon calcium imaging and whole-cell patch clamp recordings in the prelimbic area of the PFC in awake mice, in combination with computational modeling, we found that soluble human Aβ peptide inactivates α7 nAChRs, leading to increased PV-expressing interneuron activity and impaired inhibitory control over local circuits. This results in heightened and dysregulated network activity, a phenomenon increasingly recognized as an early component of AD. Specifically, we provided a detailed analysis of the neuronal activity changes over time in PFC pyramidal neurons in wild-type (WT) and knock-out (KO) mice for the different nAChRs subunits in the presence of the soluble form of Aβ peptide. In WT mice, there was an important increase in pyramidal neuron activity over time when the Aβ accumulates, something that was also observed in α5 and β2 KO mice, whereas not in α7 KO animals, implying a potentially protective role of the α7 KO. In addition, we measured the functional consequences of the human Aβ peptide in the three main classes of interneurons, the PV, SST and VIP. Only the α7 expressing PV interneurons showed a remarkable increase in activity in early stages of AD, whereas the basal GABAergic and glutamatergic neurotransmission were not altered at this early timepoint, as shown in electrophysiology experiments. By combining optogenetics and computational modeling, we then showed that while α7 nAChRs are substantially occluded by Aβ, the heteropentameric α5 and β2-containing nAChRs are only partially inactivated and thus available as therapeutic targets (Figure 2). In addition, we demonstrated that galantamine, an approved acetylcholine-esterase inhibitor (AChE-I), which acts as a PAM of α5-containing nAChRs at low concentrations, reduces neuronal hyperactivity (Koukouli et al., 2026). Accordingly, a recent study which performed an analysis of a large cohort of AD patients, investigated whether AChE-Is are associated with slower cognitive decline in AD and decreased risk of dementia. Galantamine was the only AChE-I demonstrating a significant reduction in the risk of developing severe dementia (Xu et al., 2021). This is a significant shift in perspective. It suggests that the therapeutic efficacy of galantamine may stem not solely from increased ACh levels, but from α5 receptor-specific modulation. Such a mechanism may account for the modest benefits observed in some patients and highlights the need for next-generation drugs that target specific cholinergic receptor subtypes directly, rather than boosting global ACh levels. Enhancing α5 signaling with PAMs provides more clinical relevance. Moreover, such targeted interventions could be deployed earlier in the disease course, potentially slowing progression rather than merely alleviating symptoms.

In addition, the sparing of α5 nAChRs by Aβ suggests that these receptors are structurally or spatially less vulnerable, making them viable pharmacological targets for early intervention. Future studies should explore whether enhancing α5 signaling could restore inhibitory tone and reduce hyperexcitability in other brain regions, such as the hippocampus and entorhinal cortex. By focusing on modulating specific nAChR subtypes that remain functional in the early stages of AD, we have recently suggested that by enhancing α5 nAChR activity or blocking β2, it may be possible to restore local circuit balance (Koukouli et al., 2026). The β2 blocking hypothesis is supported by a recent study which showed that hippocampal-dependent memory was altered in early AD, but it was unaffected in β2 KO mice (Sabec et al., 2025). This is in accordance with recent computational modeling predictions, which indicate that blockade of β2 receptors, leads to normalization of pyramidal neuron activity in early AD (Koukouli et al., 2026).

The data also call attention to the importance of timing in therapeutic intervention. Because α7 nAChRs appear to be selectively and substantially disrupted early in AD, therapies designed to protect or enhance their function may only be effective during a narrow window of early-stage pathology. Biomarkers that indicate specific receptor involvement, via PET imaging, cerebrospinal fluid (CSF) profiling, or EEG-based functional readouts, could be critical in identifying the right patients at the right time. Advances in PET radiochemistry and the development of specific radiotracers have allowed the detection of α7 nAChRs in the living brain. For example, despite the challenges linked to low density of nAChRs, specificity and the difficulty of ensuring adequate brain penetration, a number of efficient radioligands, such as ^11^C-(R)-MeQAA, ^11^C-NS14492 and ^18^F-ASEM, have shown potential for studying α7 nAChR distribution and density *in vivo* (Magnussen, 2025; Fontana et al., 2023). These radioligands consist important tools for understanding the role of nAChRs in AD and schizophrenia.

### Role of nicotine in health and disease

Nicotine acts as an agonist of nAChRs and exerts profound effects on the central nervous system, especially through the α4β2 and α7 subtypes. Brain imaging studies have shown that acute nicotine administration enhances activity in several brain regions, including the PFC, thalamus, and visual system, consistent with the activation of corticobasal ganglia–thalamic circuits (Brody, 2006). In addition, activation of nAChRs in the brain by nicotine or acetylcholine induces the release of various neurotransmitters such as dopamine, glutamate, serotonin, norepinephrine, and γ-aminobutyric acid (GABA; Benowitz, 2009; Picciotto et al., 1998; Pidoplichko et al., 2013; Townsend et al., 2016).

In healthy individuals, transdermal nicotine administration enhances cognitive functions such as attention and alertness, though not memory, in non-smoking adults (Barr et al., 2008; Majdi et al., 2021). Moreover, in non-smoking patients with cognitive impairment, transdermal nicotine improved both attention and memory (Barr et al., 2008; Newhouse et al., 2012; White and Levin, 2004). Consistently, preclinical studies have shown that acute nicotine administration counteracts memory and learning impairments induced by sleep deprivation in rats (Aleisa et al., 2011). Additionally, nicotine metabolites such as cotinine and nornicotine demonstrated procognitive effects without significant side effects, although these effects were more robust with cotinine (Majdi et al., 2019). The metabolites of nicotine have lower affinity than nicotine to nAChR and they interact with the receptors as type 1 PAM (Takeshima et al., 2007). Although nornicotine has the same addictive characteristics as nicotine, cotinine has been found to reduce depressive-like behavior, working-memory deficits and synaptic loss linked to chronic stress without the adverse effects (Grizzell and Echeverria, 2015). Moreover, it was found that cotinine improves visual recognition memory while decreasing tau phosphorylation in an AD mouse model (Grizzell et al., 2017). The extent of nicotine’s cognitive-enhancing properties remains controversial, given the potential contribution of active metabolites to mechanisms underlying learning and memory (Barreto et al., 2015; Gjedde, 2023). In fact, the cotinine metabolite is suggested to act through the α7 nAChR subtype, modulating both the immune system and the central nervous system, leading to decreased inflammation and enhanced neuronal signaling pathways underlying cognitive processes and neurogenesis (Echeverria et al., 2023).

In pathological contexts, nicotine shows a dual role in both neurodegenerative diseases, such as Alzheimer’s and Parkinson’s diseases and psychiatric disorders, such as schizophrenia. Several reviews have highlighted changes in nAChRs expression across these disorders underlying the involvement of α4β2,α7 and α5 subunits in cognitive impairments (Guan, 2024; Koukouli and Changeux, 2020). Recent evidence suggests that cigarette smoking is associated with a reduced risk of Parkinson’s disease (PD) incidence, supporting a potential protective effect of nicotine-related mechanisms (Rose et al., 2024). Consistently, multiple studies using experimental models of PD have demonstrated nicotine-mediated neuroprotection, notably through attenuation of α-synuclein–induced pathology (Fares et al., 2023; Hong et al., 2009; Ullah et al., 2024). These effects are thought to involve modulation of nicotinic α7 and α4β2 receptor–dependent signaling pathways, particularly within dopaminergic neurons, leading to improved neuronal survival and synaptic function (Jurado-Coronel et al., 2019; Liu et al., 2012; Sérrière et al., 2015; Huang et al., 2011; Fares et al., 2023). Notably, pharmacological activation of α7 nAChRs using the selective agonist GTS-21 has been shown to mitigate dopaminergic neuronal loss, suppress microglial activation, and reduce pro-inflammatory gene expression in PD models, further highlighting the therapeutic potential of subtype-specific nicotinic modulation in Parkinson’s disease (Piovesana et al., 2021; Park et al., 2022; Lin et al., 2025). Furthermore, both preclinical and clinical findings show that therapeutic modulation of α4β2 and α7 receptors with agonists such as nicotine or partial agonists has produced promising effects on cognition in Alzheimer’s disease and schizophrenia patients (Jourdan et al., 2020; Koukouli et al., 2017; Lv et al., 2023; Magnussen, 2025). Furthermore, major-genome wide association studies have identified a human polymorphism in the α5 nAChR gene (rs16969968) that is associated with increased susceptibility to schizophrenia (Schizophrenia Working Group of the Psychiatric Genomics Consortium, 2014; Trubetskoy et al., 2022). We have shown before that chronic nicotine administration induces pyramidal cell hyperactivity in the PFC of awake mice (Koukouli et al., 2017). In contrast, mice carrying this human α5-nicotinic subunit polymorphism (α5SNP mice), which predisposes to nicotine addiction and schizophrenia, show reduced pyramidal neuron activity in layers II/III of the prelimbic cortex compared to wild-type controls, reflecting an altered excitation/inhibition (E/I) balance. Remarkably, chronic nicotine administration was sufficient to rescue this hypofrontality phenotype, providing a mechanistic explanation for smoking as a potential form of self-medication in individuals with schizophrenia, given the higher rates of nicotine use in this population. Consistently, human carriers of the α5SNP display reduced resting-state functional connectivity and disrupted prefrontal circuitry (Hong et al., 2011).

While nicotine holds promise as a cognitive enhancer and neuroprotective agent, its therapeutic potential is limited by its addictive properties, receptor desensitization with chronic exposure, cardiovascular toxicity, and systemic side effects. Current research increasingly focuses on safer alternatives and metabolites, such as cotinine, that preserve cognitive benefits while offering a superior safety profile, particularly in models of neuroinflammation and aging (Echeverria et al., 2023; Grizzell and Echeverria, 2015; Grizzell et al., 2017).

### Role of adolescent nicotine exposure in neurocircuitry changes

Adolescence represents a transitional phase marked by profound neurobiological, behavioral, and social transformations. In humans, this developmental period typically spans from 10 to 24 years of age (Sawyer et al., 2018; Yuan et al., 2015), while in rodents it roughly ranges from postnatal day 23 to 60 (Arellano et al., 2024; Yuan et al., 2015; Reynolds et al., 2024). This stage constitutes a critical window for brain maturation, characterized by extensive synaptic pruning, progressive myelination, and refinement of neuronal circuitry, particularly within neuromodulatory systems governed by acetylcholine and dopamine, which are central to cognitive control, emotion regulation, and rewards processing (Reynolds et al., 2025, 2024). Within this framework, we propose that during adolescence nAChRs play a pivotal role in shaping adolescent synaptic plasticity and excitation-inhibition (E/I) balance, positioning this developmental period as extremely sensitive to perturbations of cholinergic signaling. Consequently, exogenous nicotine exposure during this window can deeply reshape nAChR function and downstream circuit organization, with lasting effects that extend into adulthood.

Tobacco smoking is notably prevalent among individuals under 18 years of age and is widely recognized as a gateway to subsequent substance use (Le Foll et al., 2022). Nicotine exposure during adolescence poses a significant risk factor for the later emergence of psychiatric conditions, including substance use disorders, anxiety, depression, and schizophrenia (Brown et al., 1996; Laviolette, 2021; Reynolds et al., 2025). Chronic exposure to nicotine during this period induces persistent neuroadaptations within mesocorticolimbic and prefrontal networks, regions integral to motivation, attention, and executive function (Reynolds et al., 2025; Yuan et al., 2015). These findings suggest that adolescent nicotine exposure interferes with nAChRs dependent circuits maturation, thereby increasing vulnerability to long-term cognitive and affective dysfunction.

Clinical studies have reported associations between adolescent nicotine consumption and the onset of several psychiatric disorders, particularly major depressive disorder (MDD; Brown et al., 1996). However, the causal relationship remains unresolved, emphasizing the need for longitudinal studies to better delineate the temporal link between adolescent smoking and later neurocognitive decline (Elatfy et al., 2024). Neuroimaging investigations further reveal structural brain alterations, including reductions in both gray and white matter volumes in young adolescent smokers (Peng et al., 2017).

Preclinical models have provided mechanistic insights into these observations. Nicotine exposure during adolescence disrupts multiple neurotransmitter systems, including glutamatergic, dopaminergic, GABAergic, and cholinergic signaling within the PFC, ventral tegmental area (VTA), and nucleus accumbens. These alterations heighten rewards sensitivity and increase susceptibility to addiction and long-term cognitive deficits (Counotte et al., 2009; Dao et al., 2011; Hudson et al., 2021; Jobson et al., 2019; Ng et al., 2024; Shram et al., 2007; Thomas et al., 2018; Reynolds et al., 2024). Importantly, adolescent nicotine intake interferes with the maturation of prefrontal circuits, impairing inhibitory control and synaptic plasticity, key processes critically dependent on nAChRs signaling, underlying learning and cognitive flexibility, core features underlying vulnerability to addiction and affective behavior (Goriounova and Mansvelder, 2012b).

Experimental evidence shows that repeated nicotine exposure in adolescent, but not adult rats reduce spike timing–dependent plasticity (STDP) in layer V pyramidal neurons of the PFC, mimicking the effects of direct nAChR activation (Goriounova and Mansvelder, 2012a). This reduction in STDP is attributed to an increased inhibitory tone driven by the activation of cortical interneurons through distinct α7 and β2-containing nAChR subtypes (Couey et al., 2007; Poorthuis et al., 2013). These findings underscore the importance of nAChR-mediated regulation of E/I balance during adolescent circuits refinement.

Moreover, nicotine exposure during adolescence induces long-lasting behavioral and cellular consequences. For instance, adolescent mice exposed to nicotine exhibit depressive-like behaviors in adulthood, which have been linked to impaired hippocampal neurogenesis and reduced microglial populations in the dentate gyrus, effects reversible through microglial stimulation (Fang et al., 2025), suggesting that adolescent nicotinic exposure not only alters neuronal circuitry but also engage neuroimmune pathways that contribute to long-term vulnerability.

At the molecular level, chronic nicotine administration during adolescence induces long-lasting alteration in nAChRs expression and function (Reynolds et al., 2025; Doura et al., 2008; Cano et al., 2020). Prolonged nicotine alters gene expression profiles in the prefrontal cortex, particularly genes involved in mitochondrial respiratory chain function in both pyramidal and inhibitory neurons (Yang et al., 2017). This differential gene modulation may account for nicotine’s distinct effects across neuronal subtypes. In addition, prolonged nicotine exposure in adolescent male rats leads to persistent, sex-specific changes in nAChR subunits expression, particularly affecting α7 and β2-containing nAChR receptors in the PFC, showing a significative reduction of both nicotinic receptor subunits in male but not in female rats, thereby disrupting cholinergic tone and E/I balance within cortical microcircuits (Ng et al., 2024). These receptor-level alterations result in heightened cortical excitability and aberrant neuronal oscillations (Hudson et al., 2021; Jobson et al., 2019; Ng et al., 2024). Given the established roles of α7 nAChRs in synaptic plasticity, interneuron regulation, mitochondrial signaling, and neuroinflammation (Townsend et al., 2016; ElNebrisi et al., 2025), and of β2-containing nAChRs in dopaminergic modulation and rewards processing (Grieder et al., 2019; Jiang et al., 2025; Tapper et al., 2004), these subtype-specific adaptations likely represent a mechanistic link between early nicotine exposure and enduring circuit dysfunction.

Collectively, these molecular, synaptic, and circuit-level adaptations indicate that nicotine exposure during adolescence, acts as developmental modifier of nAChRs signaling, inducing long-lasting neurobiological changes. By altering nAChRs dependent neurotransmission, synaptic plasticity, receptor expression, mitochondrial function, and neuroimmune signaling, adolescent nicotine exposure establishes a developmental vulnerability, unmasked by later life stressor, aging and neurodegenerative processes increasing the risk for cognitive impairments, affective disturbances, and substance use disorders later in life (Reynolds et al., 2025; Govind et al., 2009; Mahajan et al., 2021; Ren and Lotfipour, 2019). From a translational perspective, these findings have strong therapeutic implications, suggesting that strategies targeting nAChRs receptors, particularly those implicating subtype-specific modulation, must first, consider the developmental stage, as non-selective modulation of nAChRs may reinforce maladaptive plasticity. Second, prior nicotine exposure history should be taken into account to restore circuit function without exacerbating maladaptive circuitry. Finally, these findings argue for subtype-specific targeting approaches, such as the selective modulation of α7 or β2-containing nAChRs, aimed at restoring physiological cholinergic tone and circuit balance rather than broadly stimulating nicotinic signaling.

## Discussion

In this perspective, we explore the involvement of nAChRs in cognitive processes and discuss their promise as therapeutic targets for psychiatric and neurodegenerative disorders, such as schizophrenia and Alzheimer’s disease, respectively. We emphasize the need for subtype-specific interventions and personalized treatment strategies based on genetic variability in nicotinic genes.

### Subtype-specific nicotinic ligands: toward precision pharmacotherapy

Current challenges in nAChR pharmacotherapy increasingly point toward a shift from broad cholinergic modulation to precision medicine approaches that target specific receptor subtypes and neuronal populations. Clinical investigations have repeatedly implicated the cholinergic system in human cognitive processes, although the magnitude and consistency of these effects remain variable. Nicotinic acetylcholine receptors continue to be regarded as promising therapeutic targets for the treatment of schizophrenia and several neurodegenerative disorders. Recently, a novel pharmacological treatment for schizophrenia that targets muscarinic receptors has received FDA approval (Kingwell, 2024). This therapy is based on xanomeline, an agonist of the M1 and M4 muscarinic receptor subtypes, and produces antipsychotic effects comparable to those of existing dopaminergic antipsychotics (Shannon et al., 2000). Xanomeline exerts its effects on midbrain dopaminergic neurons indirectly, via modulation of the cholinergic inputs they receive. Importantly, the innovation of this newly approved treatment does not lie in the use of xanomeline itself, which has long been recognized for its antipsychotic properties (Bymaster et al., 1998) and shown to reduce psychotic symptoms in patients with Alzheimer’s disease (Shannon et al., 2000), but rather in its combination with trospium chloride, a peripherally restricted muscarinic antagonist. This association mitigates the well-known gastrointestinal side effects that have previously limited the development of xanomeline as a viable antipsychotic drug. These findings highlight an existing interest in the role of the cholinergic system in schizophrenia.

In particular, attention to nicotinic receptors has been driven by large-scale genome-wide association studies identifying polymorphisms in genes encoding muscarinic and nicotinic acetylcholine receptors that confer increased risk for schizophrenia (Schizophrenia Working Group of the Psychiatric Genomics Consortium, 2014; Trubetskoy et al., 2022). Nicotinic acetylcholine receptors have long occupied a central position in schizophrenia research (Leonard et al., 1996), especially in light of extensive evidence linking smoking behavior to schizophrenia (de Leon and Diaz, 2005).

Over the past two decades, multiple compounds exhibiting relative selectivity for distinct nAChR subtypes, most notably α4β2 and α7, have been developed and advanced into late preclinical testing and clinical trials (Horenstein et al., 2008; Kitagawa et al., 2003; Davidson et al., 2021; Callahan et al., 2014). Among these, pharmacological modulation of α7 nAChRs has received particular attention. Clinical studies have demonstrated that higher doses of anabaseine derivatives, such as DMXB-A, a partial α7 agonist, improve cognitive functions including attention, vigilance, and working memory in patients with schizophrenia (Freedman et al., 2008; Olincy et al., 2006). These effects parallel those observed in healthy individuals (Kitagawa et al., 2003) and have been replicated in preclinical models of schizophrenia (Callahan et al., 2014).

Beyond orthosteric agonists, positive allosteric modulators (PAMs) of α7 receptors, such as AVL-3288, have emerged as alternative strategies to enhance cognitive function while potentially minimizing receptor desensitization (Kantrowitz et al., 2020; Titus et al., 2019). Similarly, other selective α7 agonists, including encenicline (EVP-6124) and TC-5619, have demonstrated procognitive effects in schizophrenia and Alzheimer’s disease models, although clinical outcomes have been variable due to severe gastrointestinal side effects (Barbier et al., 2015; Brannan, 2019; Mazurov et al., 2012). As a result, the therapeutic efficacy of selectively targeting α7 receptors remains an area of ongoing debate (Walling et al., 2016). In parallel, α4β2-containing nAChRs have been explored as pharmacological targets, particularly in the context of cognitive impairment and smoking cessation in schizophrenia. Varenicline, a partial α4β2 agonist, has shown beneficial effects on cognition and nicotine dependence in several clinical studies (Shim et al., 2012; Smith et al., 2016; Johnstone et al., 2022). Comprehensive overviews of emerging nicotinic modulators are available elsewhere (Magnussen, 2025).

In contrast to α7 and α4β2 receptors, ligands that selectively target α5-containing (α5*) nAChRs are currently unavailable. This limitation largely arises from the unique role of the α5 subunit, which does not contribute directly to the canonical agonist-binding site but instead acts as an accessory subunit that regulates receptor assembly, trafficking, calcium permeability, and desensitization kinetics (Kuryatov et al., 2008, 2011; Scholze and Huck, 2020). These properties complicate conventional ligand-based targeting strategies. Notably, galantamine classically characterized as an acetylcholinesterase inhibitor has been shown at low concentrations to function as a positive allosteric modulator of α5-containing nAChRs (Poorthuis et al., 2013; Yang et al., 2025). Recent evidence suggests that galantamine can modulate α5 polymorphisms associated with schizophrenia and nicotine dependence, thereby opening new therapeutic avenues focused on nicotinic receptor gene dysfunction rather than receptor subtype alone (Koukouli et al., 2017; Yang et al., 2025). We recently showed that galantamine can reduce pyramidal neuron activity in a mouse model of local amyloid pathology which is characterized by PFC neuronal hyperactivity (Koukouli et al., 2026). Several clinical studies have evaluated galantamine as a cognitive enhancer in schizophrenia and dementia, reporting meaningful benefits (Deutsch et al., 2008; Xu et al., 2021). Together, these studies on galantamine suggest that allosteric modulators, which do not directly interfere with ligand binding, may represent promising targets for drug development. A major limitation in this process is the challenge of generating isolated receptor subunits that can nonetheless assemble into functional nAChRs. One approach to overcome this issue involves the use of concatemers, long continuous DNA constructs containing multiple copies of the same sequence, which promote subunit assembly through enforced proximity following translation. This strategy enabled the evaluation of galantamine efficacy in functional nAChRs (Prevost et al., 2020). More broadly, the concatemeric approach may offer a versatile platform for the efficient testing of novel compounds.

Within this framework, precision medicine approaches based on genetic stratification offer a complementary strategy to traditional ligand development. While polymorphisms in *CHRNA5* are well established in their associations with schizophrenia, nicotine dependence, and cognitive deficits, relatively few robust examples currently link specific nAChR genetic variants to differential therapeutic responses (Koukouli et al., 2017, 2026; Kuryatov et al., 2011; Liu et al., 2013; Maskos, 2020; Sinkus et al., 2015). This gap likely reflects the polygenic architecture of psychiatric disorders, incomplete functional characterization of many nAChR variants, and the limited incorporation of genetic stratification into clinical trial design (Schizophrenia Working Group of the Psychiatric Genomics Consortium, 2014; Smoller et al., 2019; Trubetskoy et al., 2022). Nevertheless, as large-scale genomic resources and functional studies continue to expand, precision medicine holds substantial promise for guiding nicotinic-based therapies tailored to patient subgroups defined by distinct nAChR genetic or expression profiles.

## Future directions and conclusion

Emerging evidence highlights distinct roles of α 5-, α 7-, and β2-containing nAChRs in cognition, memory, and neuronal circuits. The detailed structural and functional identification of the multiple conformational states of nAChRs and its homologs at the atomic level paves the way for the development of a new conformation-specific pharmacology which can result in the design of novel therapeutic agents (Cecchini et al., 2024; Avstrikova et al., 2025). Future studies should focus on developing highly selective agonists, antagonists, or PAMs that target individual receptor subtypes, particularly those preserved in disease states such as the α5 nAChRs in early Alzheimer’s disease. Subtype-specific interventions may help restore E/I balance in cortical and hippocampal circuits without the broad side effects associated with global cholinergic modulation. The differential expression of nAChRs across interneuron subtypes and cortical layers further suggests that targeting receptors in a cell-type-specific manner could optimize cognitive outcomes. Recent advances enable subtype- and circuit-specific modulation of nAChRs, overcoming limitations of traditional cholinergic approaches. Optogenetic and chemogenetic tools allow cell type resolved interrogation of receptor function and circuit dynamics (Tapper et al., 2004; Tochitsky et al., 2012; Paoletti et al., 2019). In parallel, structure-guided ligand design and allosteric modulation have improved subtype selectivity and reduced off-target effects (Durand-de Cuttoli et al., 2018; Colleoni et al., 2025; Jehl et al., 2025). Combined with genetic and molecular profiling to define therapeutic windows, these strategies support a shift toward precision, circuit-informed nAChR targeting for neuropsychiatric and neurodegenerative disorders (Shafiee et al., 2024; Fernández-Cabello et al., 2020; Ciscato et al., 2025).

Given that α7 nAChRs are selectively impaired early in Alzheimer’s pathology, identifying receptor-specific biomarkers will be critical to determine optimal therapeutic windows. Early receptor-targeted interventions aimed at heteropentameric nAChRs such as the α5, which become partially inactivated in the early stages of Alzheimer’s disease, may prevent cortical hyperexcitability and slow cognitive decline more effectively than symptomatic treatments. While nicotine itself exerts cognitive-enhancing and neuroprotective effects, its addictive potential and systemic side effects limit clinical use. Future research should therefore prioritize safer alternatives, such as metabolites, partial agonists, or novel PAMs, which preserve cognitive benefits with minimal toxicity. Testing these compounds in models of aging and psychiatric disorders may broaden their therapeutic potential. Could combining low-dose allosteric modulators of α5-containing nAChRs with anti-amyloid therapies (e.g., monoclonal antibodies) offer synergistic benefits? This integrated strategy might address both pathological protein accumulation and neuromodulatory dysfunction simultaneously.

Genetic variations, including the α5SNP associated with schizophrenia and nicotine dependence and the human specific duplication *CHRFAM7A*, significantly influence cortical excitability and receptor function. Personalized strategies that account for such genetic factors could enable tailored therapies to restore circuit balance and provide insights into mechanisms underlying self-medication behaviors in psychiatric populations. Future work should systematically assess how modulation of distinct nAChR subtypes affects multiple cognitive domains within unified experimental frameworks. Such integrative approaches could uncover convergent mechanisms linking cholinergic signaling to cognitive resilience and disease vulnerability.

## Data Availability

Publicly available datasets were analyzed in this study. This data can be found here: https://celltypes.brain-map.org/rnaseq/mouse/v1-alm.

## References

[B1] Abbondanza A. Urushadze A. Alves-Barboza A. R. Janickova H. (2024). Expression and function of nicotinic acetylcholine receptors in specific neuronal populations: Focus on striatal and prefrontal circuits. *Pharmacol. Res.* 204:107190. 10.1016/j.phrs.2024.107190 38704107

[B2] Addy N. A. Nakijama A. Levin E. D. (2003). Nicotinic mechanisms of memory: Effects of acute local DHbetaE and MLA infusions in the basolateral amygdala. *Brain Res. Cogn. Brain Res.* 16 51–57. 10.1016/s0926-6410(02)00209-4 12589888

[B3] Albuquerque E. X. Pereira E. F. Alkondon M. Rogers S. W. (2009). Mammalian nicotinic acetylcholine receptors: From structure to function. *Physiol. Rev.* 89 73–120. 10.1152/physrev.00015.2008 19126755 PMC2713585

[B4] Aleisa A. M. Helal G. Alhaider I. A. Alzoubi K. H. Srivareerat M. Tran T. T. et al. (2011). Acute nicotine treatment prevents REM sleep deprivation-induced learning and memory impairment in rat. *Hippocampus* 21 899–909. 10.1002/hipo.20806 20865738

[B5] Arellano J. I. Duque A. Rakic P. (2024). A coming-of-age story: Adult neurogenesis or adolescent neurogenesis in rodents? *Front. Neurosci.* 18:1383728. 10.3389/fnins.2024.1383728 38505771 PMC10948509

[B6] Avale M. E. Chabout J. Pons S. Serreau P. De Chaumont F. Olivo-Marin J. C. et al. (2011). Prefrontal nicotinic receptors control novel social interaction between mice. *FASEB J.* 25 2145–2155. 10.1096/fj.10-178558 21402717

[B7] Avstrikova M. Milán Rodríguez P. Burke S. M. Hibbs R. E. Changeux J. P. Cecchini M. (2025). Hidden complexity of α7 nicotinic acetylcholine receptor desensitization revealed by MD simulations and Markov state modeling. *Proc. Natl. Acad. Sci. U. S. A.* 122:e2420993122. 10.1073/pnas.2420993122 39946538 PMC11848294

[B8] Bailey C. D. De Biasi M. Fletcher P. J. Lambe E. K. (2010). The nicotinic acetylcholine receptor alpha5 subunit plays a key role in attention circuitry and accuracy. *J. Neurosci.* 30 9241–9252. 10.1523/JNEUROSCI.2258-10.2010 20610759 PMC3004929

[B9] Ballinger E. C. Ananth M. Talmage D. A. Role L. W. (2016). Basal forebrain cholinergic circuits and signaling in cognition and cognitive decline. *Neuron* 91 1199–1218. 10.1016/j.neuron.2016.09.006 27657448 PMC5036520

[B10] Barbier A. J. Hilhorst M. Van Vliet A. Snyder P. Palfreyman M. G. Gawryl M. et al. (2015). Pharmacodynamics, pharmacokinetics, safety, and tolerability of encenicline, a selective α7 nicotinic receptor partial agonist, in single ascending-dose and bioavailability studies. *Clin. Ther.* 37 311–324. 10.1016/j.clinthera.2014.09.013 25438724

[B11] Barr R. S. Culhane M. A. Jubelt L. E. Mufti R. S. Dyer M. A. Weiss A. P. et al. (2008). The effects of transdermal nicotine on cognition in nonsmokers with schizophrenia and nonpsychiatric controls. *Neuropsychopharmacology* 33 480–490. 10.1038/sj.npp.1301423 17443126

[B12] Barreto G. E. Iarkov A. Moran V. E. (2015). Beneficial effects of nicotine, cotinine and its metabolites as potential agents for Parkinson’s disease. *Front. Aging Neurosci.* 6:340. 10.3389/fnagi.2014.00340 25620929 PMC4288130

[B13] Bartus R. T. (2000). On neurodegenerative diseases, models, and treatment strategies: Lessons learned and lessons forgotten a generation following the cholinergic hypothesis. *Exp. Neurol.* 163 495–529. 10.1006/exnr.2000.7397 10833325

[B14] Benowitz N. L. (2009). Pharmacology of nicotine: Addiction, smoking-induced disease, and therapeutics. *Annu. Rev. Pharmacol. Toxicol.* 49 57–71. 10.1146/annurev.pharmtox.48.113006.094742 18834313 PMC2946180

[B15] Brannan S. (2019). 32.2 Two global phase iii trials of encenicline for cognitive impairment in chronic schizophrenia patients: Red flags and lessons learned. *Schizophrenia Bull.* 45 (Suppl. 2), S141–S142. 10.1093/schbul/sbz022.133

[B16] Brody A. L. (2006). Functional brain imaging of tobacco use and dependence. *J. Psychiatr. Res.* 40 404–418. 10.1016/j.jpsychires.2005.04.012 15979645 PMC2876087

[B17] Brown R. A. Lewinsohn P. M. Seeley J. R. Wagner E. F. (1996). Cigarette smoking, major depression, and other psychiatric disorders among adolescents. *J. Am. Acad. Child Adolesc. Psychiatry* 35 1602–1610. 10.1097/00004583-199612000-00011 8973066

[B18] Bruszt N. Bali Z. K. Nagy L. V. Bodó K. Engelmann P. Hernádi I. (2025). Synergistic effects of memantine and alpha7 nicotinic acetylcholine receptor agonist PHA-543613 to improve memory of aged rats. *Int. J. Neuropsychopharmacol.* 28:yaf014. 10.1093/ijnp/pyaf014 39985181 PMC11929953

[B19] Burns L. H. Pei Z. Wang H. Y. (2023). Targeting α7 nicotinic acetylcholine receptors and their protein interactions in Alzheimer’s disease drug development. *Drug Dev. Res.* 84 1085–1095. 10.1002/ddr.22085 37291958

[B20] Bymaster F. P. Shannon H. E. Rasmussen K. Delapp N. W. Mitch C. H. Ward J. S. et al. (1998). Unexpected antipsychotic-like activity with the muscarinic receptor ligand (5R,6R)6-(3-Propylthio-1,2,5-Thiadiazol-4-Yl)-1-Azabicyclo[3.2.1]Octane. *Eur. J. Pharmacol.* 356 109–119. 10.1016/S0014-2999(98)00487-7 9774240

[B21] Callahan P. M. Terry A. V. Tehim A. (2014). Effects of the nicotinic α7 receptor partial agonist GTS-21 on NMDA-glutamatergic receptor related deficits in sensorimotor gating and recognition memory in rats. *Psychopharmacology* 231 3695–3706. 10.1007/s00213-014-3509-2 24595504 PMC4748388

[B22] Cano M. Reynaga D. D. Belluzzi J. D. Loughlin S. E. Leslie F. (2020). Chronic exposure to cigarette smoke extract upregulates nicotinic receptor binding in adult and adolescent rats. *Neuropharmacology* 181:108308. 10.1016/j.neuropharm.2020.108308 32950561 PMC7655523

[B23] Cecchini M. Corringer P. J. Changeux J. P. (2024). The nicotinic acetylcholine receptor and its pentameric homologs: Toward an allosteric mechanism of signal transduction at the atomic level. *Annu. Rev. Biochem.* 93 339–366. 10.1146/annurev-biochem-030122-033116 38346274

[B24] Ciscato M. Stenbolt K. Mourot A. Guyon N. (2025). Covalent anchoring of tethered ligands to chemogenetic handles for targeted *in vivo* neuropharmacology. *STAR Protoc.* 6:104186. 10.1016/j.xpro.2025.104186 41223059 PMC12657727

[B25] Colleoni A. Galli G. Dallanoce C. De Amici M. Gorostiza P. Matera C. (2025). Light-Activated pharmacological tools for exploring the cholinergic system. *Med. Res. Rev.* 45 1251–1274. 10.1002/med.22108 40123150 PMC12131668

[B26] Couey J. J. Meredith R. M. Spijker S. Poorthuis R. B. Smit A. B. Brussaard A. B. et al. (2007). Distributed network actions by nicotine increase the threshold for spike-timing-dependent plasticity in prefrontal cortex. *Neuron* 54 73–87. 10.1016/j.neuron.2007.03.006 17408579

[B27] Counotte D. S. Spijker S. Van de Burgwal L. H. Hogenboom F. Schoffelmeer A. N. De Vries T. J. et al. (2009). Long-lasting cognitive deficits resulting from adolescent nicotine exposure in rats. *Neuropsychopharmacology* 34 299–306. 10.1038/npp.2008.96 18580873

[B28] Court J. Martin-Ruiz C. Piggott M. Spurden D. Griffiths M. Perry E. (2001). Nicotinic receptor abnormalities in Alzheimer’s disease. *Biol. Psychiatry* 49 175–184. 10.1016/s0006-3223(00)01116-1 11230868

[B29] Dalley J. W. Cardinal R. N. Robbins T. W. (2004). Prefrontal executive and cognitive functions in rodents: Neural and neurochemical substrates. *Neurosci. Biobehav. Rev.* 28 771–784. 10.1016/j.neubiorev.2004.09.006 15555683

[B30] Dao J. M. McQuown S. C. Loughlin S. E. Belluzzi J. D. Leslie F. M. (2011). Nicotine alters limbic function in adolescent rat by a 5-HT1A receptor mechanism. *Neuropsychopharmacology* 36 1319–1331. 10.1038/npp.2011.8 21412223 PMC3096821

[B31] Davidson M. Levi L. Park J. Nastas I. Ford L. Rassnick S. et al. (2021). The effects of JNJ-39393406 a positive allosteric nicotine modulator on mood and cognition in patients with unipolar depression: A double-blind, add-on, placebo-controlled trial. *Eur. Neuropsychopharmacol.* 51 33–42. 10.1016/j.euroneuro.2021.04.020 34023797

[B32] Davies P. Maloney A. J. (1976). Selective loss of central cholinergic neurons in Alzheimer’s disease. *Lancet* 2:1403. 10.1016/s0140-6736(76)91936-x 63862

[B33] de Leon J. Diaz F. J. (2005). A meta-analysis of worldwide studies demonstrates an association between schizophrenia and tobacco smoking behaviors. *Schizophr. Res.* 76 135–157. 10.1016/j.schres.2005.02.010 15949648

[B34] Dere E. Huston J. P. De Souza Silva M. A. (2007). The pharmacology, neuroanatomy and neurogenetics of one-trial object recognition in rodents. *Neurosci. Biobehav. Rev.* 31 673–704. 10.1016/j.neubiorev.2007.01.005 17368764

[B35] Deutsch S. I. Rosse R. B. Schwartz B. L. Schooler N. R. Gaskins B. L. Long K. D. et al. (2008). Effects of CDP-choline and the combination of CDP-choline and galantamine differ in an animal model of schizophrenia: Development of a selective alpha7 nicotinic acetylcholine receptor agonist strategy. *Eur. Neuropsychopharmacol.* 18 147–151. 10.1016/j.euroneuro.2007.05.008 17656074

[B36] Dineley K. T. Pandya A. A. Yakel J. L. (2015). Nicotinic ACh receptors as therapeutic targets in CNS disorders. *Trends Pharmacol. Sci.* 36 96–108. 10.1016/j.tips.2014.12.002 25639674 PMC4324614

[B37] Doura M. B. Gold A. B. Keller A. B. Perry D. C. (2008). Adult and periadolescent rats differ in expression of nicotinic cholinergic receptor subtypes and in the response of these subtypes to chronic nicotine exposure. *Brain Res.* 1215 40–52. 10.1016/j.brainres.2008.03.056 18474362 PMC2493527

[B38] Durand-de Cuttoli R. Mondoloni S. Marti F. Lemoine D. Nguyen C. Naudé J. et al. (2018). Manipulating midbrain dopamine neurons and reward-related behaviors with light-controllable nicotinic acetylcholine receptors. *Elife* 7:e37487. 10.7554/eLife.37487 30176987 PMC6122951

[B39] Echeverria V. Mendoza C. Iarkov A. (2023). Nicotinic acetylcholine receptors and learning and memory deficits in Neuroinflammatory diseases. *Front. Neurosci.* 17:1179611. 10.3389/fnins.2023.1179611 37255751 PMC10225599

[B40] Elatfy A. Vrahimis S. Conti A. Baldacchino A. (2024). Chronic tobacco smoking and neurocognitive impairments in adolescents and young adults: A systematic review and meta-analysis. *Front. Psychiatry* 15:1384408. 10.3389/fpsyt.2024.1384408 38716120 PMC11074441

[B41] ElNebrisi E. Lozon Y. Oz M. (2025). The role of α7-Nicotinic acetylcholine receptors in the pathophysiology and treatment of Parkinson’s disease. *Int. J. Mol. Sci.* 26:3210. 10.3390/ijms26073210 40244021 PMC11990008

[B42] Esaki H. Izumi S. Fukao A. Ito S. Nishitani N. Deyama S. et al. (2021). Nicotine enhances object recognition memory via stimulating α4β2 and α7 nicotinic acetylcholine receptors in the medial prefrontal cortex of mice. *Biol. Pharm. Bull.* 44 1007–1013. 10.1248/bpb.b21-00314 34193682

[B43] Fang Y. Yang R. Song R. Gu Y. Lu X. Chen Z. et al. (2025). Nicotine exposure in adolescence triggers the activation and subsequent damage of microglia in the dentate gyrus and promotes depression later in life. *Int. Immunopharmacol.* 161:115060. 10.1016/j.intimp.2025.115060 40513337

[B44] Fares M. B. Alijevic O. Johne S. Overk C. Hashimoto M. Kondylis A. et al. (2023). Nicotine-mediated effects in neuronal and mouse models of synucleinopathy. *Front. Neurosci.* 17:1239009. 10.3389/fnins.2023.1239009 37719154 PMC10501483

[B45] Fernández-Cabello S. Kronbichler M. Van Dijk K. R. A. Goodman J. A. Spreng R. N. Schmitz T. W. (2020). Basal forebrain volume reliably predicts the cortical spread of Alzheimer’s degeneration. *Brain* 143 993–1009. 10.1093/brain/awaa012 32203580 PMC7092749

[B46] Fontana I. C. Kumar A. Nordberg A. (2023). The role of astrocytic α7 nicotinic acetylcholine receptors in Alzheimer disease. *Nat. Rev. Neurol.* 19 278–288. 10.1038/s41582-023-00792-4 36977843

[B47] Freedman R. Coon H. Myles-Worsley M. Orr-Urtreger A. Olincy A. Davis A. et al. (1997). Linkage of a neurophysiological deficit in schizophrenia to a chromosome 15 locus. *Proc. Natl. Acad. Sci. U. S. A.* 94 587–592. 10.1073/pnas.94.2.587 9012828 PMC19557

[B48] Freedman R. Olincy A. Buchanan R. W. Harris J. G. Gold J. M. Johnson L. et al. (2008). Initial phase 2 trial of a nicotinic agonist in schizophrenia. *Am. J. Psychiatry* 165 1040–1047. 10.1176/appi.ajp.2008.07071135 18381905 PMC3746983

[B49] Garduño J. Galindo-Charles L. Jiménez-Rodríguez J. Galarraga E. Tapia D. Mihailescu S. et al. (2012). Presynaptic α4β2 nicotinic acetylcholine receptors increase glutamate release and serotonin neuron excitability in the dorsal raphe nucleus. *J. Neurosci.* 32 15148–15157. 10.1523/JNEUROSCI.0941-12.2012 23100436 PMC6704822

[B50] Gerzanich V. Wang F. Kuryatov A. Lindstrom J. (1998). A 5 subunit alters desensitization, pharmacology, Ca^++^ permeability and Ca^++^ modulation of human neuronal *A*3 nicotinic receptors1. *J. Pharmacol. Exp. Therapeut.* 286 311–320. 10.1016/S0022-3565(24)37589-5 9655874

[B51] Gjedde A. (2023). Editorial: Nicotine and its derivatives in disorders of cognition: A challenging new topic of study. *Front. Neurosci.* 17:1252705. 10.3389/fnins.2023.1252705 37534040 PMC10393245

[B52] Görgülü I. Jagannath V. Pons S. Koniuszewski F. Groszer M. Maskos U. et al. (2024). The human-specific nicotinic receptor subunit CHRFAM7A reduces α7 receptor function in human induced pluripotent stem cells-derived and transgenic mouse neurons. *Eur. J. Neurosci.* 60 4893–4906. 10.1111/ejn.16474 39073048

[B53] Goriounova N. A. Mansvelder H. D. (2012a). Nicotine exposure during adolescence alters the rules for prefrontal cortical synaptic plasticity during adulthood. *Front. Synaptic Neurosci.* 4:3. 10.3389/fnsyn.2012.00003 22876231 PMC3410598

[B54] Goriounova N. A. Mansvelder H. D. (2012b). Nicotine exposure during adolescence leads to short- and long-term changes in spike timing-dependent plasticity in rat prefrontal cortex. *J. Neurosci.* 32 10484–10493. 10.1523/JNEUROSCI.5502-11.2012 22855798 PMC3552309

[B55] Gotti C. Zoli M. Clementi F. (2006). Brain nicotinic acetylcholine receptors: Native subtypes and their relevance. *Trends Pharmacol. Sci.* 27 482–491. 10.1016/j.tips.2006.07.004 16876883

[B56] Govind A. P. Vezina P. Green W. N. (2009). Nicotine-induced upregulation of nicotinic receptors: Underlying mechanisms and relevance to nicotine addiction. *Biochem. Pharmacol.* 78 756–765. 10.1016/j.bcp.2009.06.011 19540212 PMC2728164

[B57] Grieder T. E. Besson M. Maal-Bared G. Pons S. Maskos U. van der Kooy D. (2019). β2* nAChRs on VTA dopamine and GABA neurons separately mediate nicotine aversion and reward. *Proc. Natl. Acad. Sci. U. S. A.* 116 25968–25973. 10.1073/pnas.1908724116 31776253 PMC6925992

[B58] Grizzell J. A. Echeverria V. (2015). New insights into the mechanisms of action of cotinine and its distinctive effects from nicotine. *Neurochem. Res.* 40 2032–2046. 10.1007/s11064-014-1359-2 24970109

[B59] Grizzell J. A. Patel S. Barreto G. E. Echeverria V. (2017). Cotinine improves visual recognition memory and decreases cortical Tau phosphorylation in the Tg6799 mice. *Prog. Neuropsychopharmacol. Biol. Psychiatry* 78 75–81. 10.1016/j.pnpbp.2017.05.010 28536070

[B60] Grupe M. Paolone G. Jensen A. A. Sandager-Nielsen K. Sarter M. Grunnet M. (2013). Selective potentiation of (α4)3(β2)2 nicotinic acetylcholine receptors augments amplitudes of prefrontal acetylcholine- and nicotine-evoked glutamatergic transients in rats. *Biochem. Pharmacol.* 86 1487–1496. 10.1016/j.bcp.2013.09.005 24051136 PMC3915712

[B61] Guan Z. Z. (2024). Alterations in neuronal nicotinic acetylcholine receptors in the pathogenesis of various cognitive impairments. *CNS Neurosci. Ther.* 30:e70069. 10.1111/cns.70069 39370620 PMC11456617

[B62] Guillem K. Bloem B. Poorthuis R. B. Loos M. Smit A. B. Maskos U. et al. (2011). Nicotinic acetylcholine receptor β2 subunits in the medial prefrontal cortex control attention. *Science* 333 888–891. 10.1126/science.1207079 21836018

[B63] Hardy J. Allsop D. (1991). Amyloid deposition as the central event in the aetiology of Alzheimer’s disease. *Trends Pharmacol. Sci.* 12 383–388. 10.1016/0165-6147(91)90609-v 1763432

[B64] Hong D. P. Fink A. L. Uversky V. N. (2009). Smoking and Parkinson’s disease: Does nicotine affect alpha-synuclein fibrillation? *Biochim. Biophys. Acta* 1794 282–290. 10.1016/j.bbapap.2008.09.026 19013262 PMC2647853

[B65] Hong L. E. Yang X. Wonodi I. Hodgkinson C. A. Goldman D. Stine O. C. et al. (2011). A *CHRNA5* allele related to nicotine addiction and schizophrenia. *Genes Brain Behav.* 10 530–535. 10.1111/j.1601-183X.2011.00689.x 21418140 PMC3126887

[B66] Horenstein N. A. Leonik F. M. Papke R. L. (2008). Multiple pharmacophores for the selective activation of nicotinic alpha7-type acetylcholine receptors. *Mol. Pharmacol.* 74 1496–1511. 10.1124/mol.108.048892 18768388 PMC2999882

[B67] Hoyle E. Genn R. F. Fernandes C. Stolerman I. P. (2006). Impaired performance of alpha7 nicotinic receptor knockout mice in the five-choice serial reaction time task. *Psychopharmacology* 189 211–223. 10.1007/s00213-006-0549-2 17019565 PMC1705494

[B68] Huang L. Z. Grady S. R. Quik M. (2011). Nicotine reduces L-DOPA-induced dyskinesias by acting at beta2* nicotinic receptors. *J. Pharmacol. Exp. Ther.* 338 932–941. 10.1124/jpet.111.182949 21665941 PMC3164339

[B69] Hudson R. Green M. Wright D. J. Renard J. Jobson C. E. L. Jung T. et al. (2021). Adolescent nicotine induces depressive and anxiogenic effects through ERK 1-2 and Akt-GSK-3 pathways and neuronal dysregulation in the nucleus accumbens. *Addict. Biol.* 26:e12891. 10.1111/adb.12891 32135573

[B70] Ikonomovic M. D. Wecker L. Abrahamson E. E. Wuu J. Counts S. E. Ginsberg S. D. et al. (2009). Cortical alpha7 nicotinic acetylcholine receptor and beta-amyloid levels in early Alzheimer disease. *Arch. Neurol.* 66 646–651. 10.1001/archneurol.2009.46 19433665 PMC2841566

[B71] Ishida S. Kawasaki Y. Araki H. Asanuma M. Matsunaga H. Sendo T. et al. (2011). α7 Nicotinic acetylcholine receptors in the central amygdaloid nucleus alter naloxone-induced withdrawal following a single exposure to morphine. *Psychopharmacology* 214 923–931. 10.1007/s00213-010-2101-7 21125398

[B72] Izumi S. Kawasaki I. Waki F. Nishikawa K. Nishitani N. Deyama S. et al. (2025). Chronic nicotine enhances object recognition memory via inducing long-term potentiation in the medial prefrontal cortex in mice. *Neuropharmacology* 273:110435. 10.1016/j.neuropharm.2025.110435 40154943

[B73] Jehl J. Ciscato M. Vicq E. Guyon N. Dejean de la Batie G. Mondoloni S. et al. (2025). The interpeduncular nucleus blunts the rewarding effect of nicotine. *Neuron* 113 1898–1907.e6. 10.1016/j.neuron.2025.03.035. 40262615 PMC12181049

[B74] Ji D. Lape R. Dani J. A. (2001). Timing and location of nicotinic activity enhances or depresses hippocampal synaptic plasticity. *Neuron* 31 131–141. 10.1016/s0896-6273(01)00332-4 11498056

[B75] Jiang J. Li X. Hu A. F. Zhou G. J. Gao Y. H. Xu C. et al. (2025). Nicotine and neuronal nicotinic acetylcholine receptors: Unraveling the mechanisms of nicotine addiction. *Front. Neurosci.* 19:1670883. 10.3389/fnins.2025.1670883 41179996 PMC12575229

[B76] Jiang Y. Yuan H. Huang L. Hou X. Zhou R. Dang X. (2019). Global proteomic profiling of the uniquely human CHRFAM7A gene in transgenic mouse brain. *Gene* 714:143996. 10.1016/j.gene.2019.143996 31348980

[B77] Jobson C. L. M. Renard J. Szkudlarek H. Rosen L. G. Pereira B. Wright D. J. et al. (2019). Adolescent nicotine exposure induces dysregulation of mesocorticolimbic activity states and depressive and anxiety-like prefrontal cortical molecular phenotypes persisting into adulthood. *Cereb. Cortex* 29 3140–3153. 10.1093/cercor/bhy179 30124787

[B78] Johnstone S. Sorkhou M. Rabin R. A. George T. P. (2022). Dose-dependent effects of Varenicline on tobacco craving and withdrawal in tobacco smokers with and without schizophrenia. *Drug Alcohol Depend.* 234:109412. 10.1016/j.drugalcdep.2022.109412 35395548

[B79] Jourdan J. P. Bureau R. Rochais C. Dallemagne P. (2020). Drug repositioning: A brief overview. *J. Pharm. Pharmacol.* 72 1145–1151. 10.1111/jphp.13273 32301512 PMC7262062

[B80] Jurado-Coronel J. C. Loaiza A. E. Díaz J. E. Cabezas R. Ashraf G. M. Sahebkar A. et al. (2019). (E)-Nicotinaldehyde O-Cinnamyloxime, a nicotine analog, attenuates neuronal cells death against rotenone-induced neurotoxicity. *Mol. Neurobiol.* 56 1221–1232. 10.1007/s12035-018-1163-0 29881944

[B81] Kandel E. R. (ed.) (2013). *Principles of Neural Science*, 5th Edn. Columbus, OH: McGraw-Hill.

[B82] Kantrowitz J. T. Javitt D. C. Freedman R. Sehatpour P. Kegeles L. S. Carlson M. et al. (2020). Double blind, two dose, randomized, placebo-controlled, cross-over clinical trial of the positive allosteric modulator at the alpha7 nicotinic cholinergic receptor AVL-3288 in schizophrenia patients. *Neuropsychopharmacology* 45 1339–1345. 10.1038/s41386-020-0628-9 32015461 PMC7298033

[B83] Kingwell K. (2024). Muscarinic drugs breathe new life into schizophrenia pipeline. *Nat. Rev. Drug Discov.* 23 647–649. 10.1038/d41573-024-00129-w 39080500

[B84] Kitagawa H. Takenouchi T. Azuma R. Wesnes K. A. Kramer W. G. Clody D. E. et al. (2003). Safety, pharmacokinetics, and effects on cognitive function of multiple doses of GTS-21 in healthy, male volunteers. *Neuropsychopharmacology* 28 542–551. 10.1038/sj.npp.1300028 12629535

[B85] Koukouli F. Changeux J. P. (2020). Do nicotinic receptors modulate high-order cognitive processing? *Trends Neurosci.* 43 550–564. 10.1016/j.tins.2020.06.001 32591156

[B86] Koukouli F. Maskos U. (2015). The multiple roles of the α7 nicotinic acetylcholine receptor in modulating glutamatergic systems in the normal and diseased nervous system. *Biochem. Pharmacol.* 97 378–387. 10.1016/j.bcp.2015.07.018 26206184

[B87] Koukouli F. Rooy M. Changeux J. P. Maskos U. (2016b). Nicotinic receptors in mouse prefrontal cortex modulate ultraslow fluctuations related to conscious processing. *Proc. Natl. Acad. Sci. U. S. A.* 113 14823–14828. 10.1073/pnas.1614417113 27911815 PMC5187677

[B88] Koukouli F. Rooy M. Maskos U. (2016a). Early and progressive deficit of neuronal activity patterns in a model of local amyloid pathology in mouse prefrontal cortex. *Aging*. 8, 3430–3449. 10.18632/aging.101136 27999185 PMC5270678

[B89] Koukouli F. Rooy M. Tziotis D. Sailor K. A. O’Neill H. C. Levenga J. et al. (2017). Nicotine reverses hypofrontality in animal models of addiction and schizophrenia. *Nat. Med.* 23 347–354. 10.1038/nm.4274 28112735 PMC5819879

[B90] Koukouli F. Zhang C. L. Lazarevich I. Rooy M. Lamotte d’Incamps B. Gaspar Santos D. et al. (2026). The alpha7 nicotinic acetylcholine receptor mediates network dysfunction in a mouse model of local amyloid pathology. *Mol. Psychiatry* 31 1293–1310. 10.1038/s41380-025-03241-4 40987885

[B91] Kuryatov A. Berrettini W. Lindstrom J. (2011). Acetylcholine receptor (AChR) α5 subunit variant associated with risk for nicotine dependence and lung cancer reduces (α4β2)*2*α5 AChR function. *Mol. Pharmacol.* 79 119–125. 10.1124/mol.110.066357 20881005 PMC3014277

[B92] Kuryatov A. Onksen J. Lindstrom J. (2008). Roles of accessory subunits in alpha4beta2(*) nicotinic receptors. *Mol. Pharmacol.* 74 132–143. 10.1124/mol.108.046789 18381563

[B93] Lamb P. W. Melton M. A. Yakel J. L. (2005). Inhibition of neuronal nicotinic acetylcholine receptor channels expressed in Xenopus oocytes by beta-amyloid1-42 peptide. *J. Mol. Neurosci.* 27 13–21. 10.1385/JMN:27:1:013 16055943

[B94] Lasala M. Corradi J. Bruzzone A. Esandi M. D. C. Bouzat C. (2018). A human-specific, truncated α7 nicotinic receptor subunit assembles with full-length α7 and forms functional receptors with different stoichiometries. *J. Biol. Chem.* 293 10707–10717. 10.1074/jbc.RA117.001698 29784875 PMC6036215

[B95] Lauterborn J. C. Scaduto P. Cox C. D. Schulmann A. Lynch G. Gall C. M. et al. (2021). Increased excitatory to inhibitory synaptic ratio in parietal cortex samples from individuals with Alzheimer’s disease. *Nat. Commun.* 12:2603. 10.1038/s41467-021-22742-8 33972518 PMC8110554

[B96] Laviolette S. R. (2021). Molecular and neuronal mechanisms underlying the effects of adolescent nicotine exposure on anxiety and mood disorders. *Neuropharmacology* 184:108411. 10.1016/j.neuropharm.2020.108411 33245960

[B97] Le Foll B. Piper M. E. Fowler C. D. Tonstad S. Bierut L. Lu L. et al. (2022). Tobacco and nicotine use. *Nat. Rev. Dis. Primers* 8:19. 10.1038/s41572-022-00346-w 35332148

[B98] Leonard S. Adams C. Breese C. R. Adler L. E. Bickford P. Byerley W. et al. (1996). Nicotinic receptor function in schizophrenia. *Schizophr. Bull.* 22 431–445. 10.1093/schbul/22.3.431 8873294

[B99] Levin E. D. McClernon F. J. Rezvani A. H. (2006). Nicotinic effects on cognitive function: Behavioral characterization, pharmacological specification, and anatomic localization. *Psychopharmacology* 184 523–539. 10.1007/s00213-005-0164-7 16220335

[B100] Lin X. Li Q. Pu M. Dong H. Zhang Q. (2025). Significance of nicotine and nicotinic acetylcholine receptors in Parkinson’s disease. *Front. Aging Neurosci.* 17:1535310. 10.3389/fnagi.2025.1535310 40191787 PMC11968747

[B101] Liu Q. Kawai H. Berg D. K. (2001). beta -Amyloid peptide blocks the response of alpha 7-containing nicotinic receptors on hippocampal neurons. *Proc. Natl. Acad. Sci. U. S. A.* 98 4734–4739. 10.1073/pnas.081553598 11274373 PMC31903

[B102] Liu X. Hong X. Chan R. C. Kong F. Peng Z. Wan X. et al. (2013). Association study of polymorphisms in the alpha 7 nicotinic acetylcholine receptor subunit and catechol-o-methyl transferase genes with sensory gating in first-episode schizophrenia. *Psychiatry Res.* 209 431–438. 10.1016/j.psychres.2013.03.027 23598060

[B103] Liu Y. Hu J. Wu J. Zhu C. Hui Y. Han Y. et al. (2012). α7 nicotinic acetylcholine receptor-mediated neuroprotection against dopaminergic neuron loss in an MPTP mouse model via inhibition of astrocyte activation. *J. Neuroinflamm.* 9:98. 10.1186/1742-2094-9-98 22624500 PMC3416733

[B104] Lv J. Duan Y. Wang X. Wu H. Chen J. Zhang W. et al. (2023). Alpha7 nicotinic acetylcholine receptor agonist PHA-543613 improves memory deficits in presenilin 1 and presenilin 2 conditional double knockout mice. *Exp. Neurol.* 359:114271. 10.1016/j.expneurol.2022.114271 36370840

[B105] Lykhmus O. Tzeng W. Y. Koval L. Uspenska K. Zirdum E. Kalashnyk O. et al. (2024). Impairment of brain function in a mouse model of Alzheimer’s disease during the pre-depositing phase: The role of α7 nicotinic acetylcholine receptors. *Biomed. Pharmacother.* 178:117255. 10.1016/j.biopha.2024.117255 39116785

[B106] Ma K. G. Qian Y. H. (2019). Alpha 7 nicotinic acetylcholine receptor and its effects on Alzheimer’s disease. *Neuropeptides* 73 96–106. 10.1016/j.npep.2018.12.003 30579679

[B107] Magnussen J. H. (2025). Therapeutic targeting of the α7 nicotinic receptor: Challenges and prospects for cognitive improvement in alzheimer’s and schizophrenia. *Basic Clin. Pharmacol. Toxicol.* 137:e70061. 10.1111/bcpt.70061 40456556 PMC12129647

[B108] Mahajan S. D. Homish G. G. Quisenberry A. (2021). Multifactorial etiology of adolescent nicotine addiction: A review of the neurobiology of nicotine addiction and its implications for smoking cessation pharmacotherapy. *Front. Public Health* 9:664748. 10.3389/fpubh.2021.664748 34291026 PMC8287334

[B109] Majdi A. Kamari F. Sadigh-Eteghad S. Gjedde A. (2019). Molecular insights into memory-enhancing metabolites of nicotine in brain: A systematic review. *Front. Neurosci.* 12:1002. 10.3389/fnins.2018.01002 30697142 PMC6341027

[B110] Majdi A. Sadigh-Eteghad S. Gjedde A. (2021). Effects of transdermal nicotine delivery on cognitive outcomes: A meta-analysis. *Acta Neurol. Scand.* 144 179–191. 10.1111/ane.13436 33899218

[B111] Maskos U. (2020). The nicotinic receptor alpha5 coding polymorphism rs16969968 as a major target in disease: Functional dissection and remaining challenges. *J. Neurochem.* 154 241–250. 10.1111/jnc.14989 32078158

[B112] Mattson M. P. (2004). Pathways towards and away from Alzheimer’s disease. *Nature* 430 631–639. 10.1038/nature02621 15295589 PMC3091392

[B113] Mazurov A. A. Kombo D. C. Hauser T. A. Miao L. Dull G. Genus J. F. et al. (2012). Discovery of (2S,3R)-N-[2-(Pyridin-3-Ylmethyl)-1-Azabicyclo[2.2.2]Oct-3-Yl]Benzo[b]Furan-2-Carboxamide (TC-5619), a selective A 7 nicotinic acetylcholine receptor agonist, for the treatment of cognitive disorders. *J. Med. Chem.* 55 9793–9809. 10.1021/jm301048a 23126648

[B114] Mendez I. A. Damborsky J. C. Winzer-Serhan U. H. Bizon J. L. Setlow B. (2013). A 4β2 and α7 nicotinic acetylcholine receptor binding predicts choice preference in two cost benefit decision-making tasks. *Neuroscience* 230 121–131. 10.1016/j.neuroscience.2012.10.067 23159316 PMC3545051

[B115] Mesulam M. (2004). The cholinergic lesion of Alzheimer’s disease: Pivotal factor or side show? *Learn Mem.* 11 43–49. 10.1101/lm.69204 14747516

[B116] Miguelez Fernández A. M. M. Molla H. M. Thomases D. R. Tseng K. Y. (2021). Prefrontal α7nAChR signaling differentially modulates afferent drive and trace fear conditioning behavior in adolescent and adult rats. *J. Neurosci.* 41 1908–1916. 10.1523/JNEUROSCI.1941-20.2020 33478990 PMC7939093

[B117] Mufson E. J. Counts S. E. Perez S. E. Ginsberg S. D. (2008). Cholinergic system during the progression of Alzheimer’s disease: Therapeutic implications. *Expert Rev. Neurother.* 8 1703–1718. 10.1586/14737175.8.11.1703 18986241 PMC2631573

[B118] Nakauchi S. Sumikawa K. (2014). Endogenous ACh suppresses LTD induction and nicotine relieves the suppression via different nicotinic ACh receptor subtypes in the mouse hippocampus. *Life Sci.* 111 62–68. 10.1016/j.lfs.2014.07.014 25046735 PMC4141526

[B119] Newhouse P. Kellar K. Aisen P. White H. Wesnes K. Coderre E. et al. (2012). Nicotine treatment of mild cognitive impairment: A 6-month double-blind pilot clinical trial. *Neurology* 78 91–101. 10.1212/WNL.0b013e31823efcbb 22232050 PMC3466669

[B120] Ng T. H. J. Sarikahya M. H. Hudson R. Szkudlarek H. J. Pérez-Valenzuela E. Uzuneser T. C. et al. (2024). Adolescent nicotine exposure induces long-term, sex-specific disturbances in mood and anxiety-related behavioral, neuronal and molecular phenotypes in the mesocorticolimbic system. *Neuropsychopharmacology* 49 1171–1182. 10.1038/s41386-024-01853-y 38521861 PMC11109238

[B121] Nordberg A. Winblad B. (1986). Reduced number of [3H]Nicotine and [3H]Acetylcholine binding sites in the frontal cortex of Alzheimer brains. *Neurosci. Lett.* 72 115–120. 10.1016/0304-3940(86)90629-4 3808458

[B122] Nott A. Levin E. D. (2006). Dorsal hippocampal alpha7 and alpha4beta2 nicotinic receptors and memory. *Brain Res.* 1081 72–78. 10.1016/j.brainres.2006.01.052 16556437

[B123] Olincy A. Harris J. G. Johnson L. L. Pender V. Kongs S. Allensworth D. et al. (2006). Proof-of-concept trial of an alpha7 nicotinic agonist in schizophrenia. *Arch. Gen. Psychiatry* 63 630–638. 10.1001/archpsyc.63.6.630 16754836

[B124] Palombo P. Maeda R. Riberti Zaniboni C. Antonagi Engi S. Yokoyama T. Bonetti Bertagna N. et al. (2024). Unlocking the role of dorsal hippocampal α4β2 nicotinic acetylcholine receptors in Ethanol-Induced conditioned place preference in mice. *Neurosci. Lett.* 824:137666. 10.1016/j.neulet.2024.137666 38331019

[B125] Paoletti P. Ellis-Davies G. C. R. Mourot A. (2019). Optical control of neuronal ion channels and receptors. *Nat. Rev. Neurosci.* 20 514–532. 10.1038/s41583-019-0197-2 31289380 PMC6703956

[B126] Parikh V. Kozak R. Martinez V. Sarter M. (2007). Prefrontal acetylcholine release controls cue detection on multiple timescales. *Neuron* 56 141–154. 10.1016/j.neuron.2007.08.025 17920021 PMC2084212

[B127] Park J. E. Leem Y. H. Park J. S. Kim D. Y. Kang J. L. Kim H. S. (2022). Anti-Inflammatory and neuroprotective mechanisms of GTS-21, an α7 nicotinic acetylcholine receptor agonist, in neuroinflammation and Parkinson’s disease mouse models. *Int. J. Mol. Sci.* 23:4420. 10.3390/ijms23084420 35457238 PMC9026703

[B128] Pastor V. Medina J. H. (2024). α7 nicotinic acetylcholine receptor in memory processing. *Eur. J. Neurosci.* 59 2138–2154. 10.1111/ejn.15913 36634032

[B129] Pastor V. Castillo Díaz F. Sanabria V. C. Dalto J. F. Antonelli M. C. Medina J. H. (2021). Prefrontal cortex nicotinic receptor inhibition by methyllycaconitine impaired cocaine-associated memory acquisition and retrieval. *Behav. Brain Res.* 406:113212. 10.1016/j.bbr.2021.113212 33657437

[B130] Patel A. V. Nguyen A. Geremia A. A. Zeng D. Paletta P. Choleris E. et al. (2025). Unique nicotinic responses are present in distinct subtypes of mouse medial prefrontal layer V pyramidal neurons. *Sci. Rep.* 15:25025. 10.1038/s41598-025-10465-5 40646084 PMC12254383

[B131] Peng P. Wang Z. Jiang T. Chu S. Wang S. Xiao D. (2017). Brain-volume changes in young and middle-aged smokers: A DARTEL-based voxel-based morphometry study. *Clin. Respir. J.* 11 621–631. 10.1111/crj.12393 26404024

[B132] Pi H. J. Hangya B. Kvitsiani D. Sanders J. I. Huang Z. J. Kepecs A. (2013). Cortical interneurons that specialize in disinhibitory control. *Nature* 503 521–524. 10.1038/nature12676 24097352 PMC4017628

[B133] Picciotto M. R. Zoli M. Rimondini R. Léna C. Marubio L. M. Pich E. M. et al. (1998). Acetylcholine receptors containing the beta2 subunit are involved in the reinforcing properties of nicotine. *Nature* 391 173–177. 10.1038/34413 9428762

[B134] Pidoplichko V. I. Prager E. M. Aroniadou-Anderjaska V. Braga M. F. (2013). α7-Containing nicotinic acetylcholine receptors on interneurons of the basolateral amygdala and their role in the regulation of the network excitability. *J. Neurophysiol.* 110 2358–2369. 10.1152/jn.01030.2012 24004528 PMC3841870

[B135] Piovesana R. Salazar Intriago M. S. Dini L. Tata A. M. (2021). Cholinergic modulation of neuroinflammation: Focus on α7 nicotinic receptor. *Int. J. Mol. Sci.* 22:4912. 10.3390/ijms22094912 34066354 PMC8125157

[B136] Poorthuis R. B. Bloem B. Schak B. Wester J. de Kock C. P. Mansvelder H. D. (2013). Layer-specific modulation of the prefrontal cortex by nicotinic acetylcholine receptors. *Cereb. Cortex* 23 148–161. 10.1093/cercor/bhr390 22291029 PMC3513956

[B137] Poorthuis R. B. Muhammad K. Wang M. Verhoog M. B. Junek S. Wrana A. et al. (2018). Rapid neuromodulation of layer 1 interneurons in human neocortex. *Cell Rep.* 23 951–958. 10.1016/j.celrep.2018.03.111 29694902 PMC5946807

[B138] Prevost M. S. Bouchenaki H. Barilone N. Gielen M. Corringer P. J. (2020). Concatemers to re-investigate the role of α5 in α4β2 nicotinic receptors. *Cell. Mol. Life Sci.* 78, 1051–1064. 10.1007/s00018-020-03558-z 32472188 PMC11071962

[B139] Qi G. Yang D. Messore F. Bast A. Yáñez F. Oberlaender M. et al. (2024). FOXP2-immunoreactive corticothalamic neurons in neocortical layers 6a and 6b are tightly regulated by neuromodulatory systems. *iScience* 28:111646. 10.1016/j.isci.2024.111646 39868047 PMC11758397

[B140] Raybuck J. D. Gould T. J. (2010). The role of nicotinic acetylcholine receptors in the medial prefrontal cortex and hippocampus in trace fear conditioning. *Neurobiol. Learn. Mem.* 94 353–363. 10.1016/j.nlm.2010.08.001 20727979 PMC2949463

[B141] Ren M. Lotfipour S. (2019). Nicotine gateway effects on adolescent substance use. *West J. Emerg. Med.* 20 696–709. 10.5811/westjem.2019.7.41661 31539325 PMC6754186

[B142] Rennie K. (2025). Neuronal and Glial α7 nicotinic acetylcholine receptors: Role in Alzheimer’s disease pathophysiology. *Life* 15:1032. 10.3390/life15071032 40724534 PMC12298375

[B143] Reynolds L. M. Faure P. Barik J. (2025). Adolescent nicotine exposure and persistent neurocircuitry changes: Unveiling lifelong psychiatric risks. *Mol. Psychiatry* 30 5534–5545. 10.1038/s41380-025-03110-0 40883452 PMC12532584

[B144] Reynolds L. M. Gulmez A. Fayad S. L. Campos R. C. Rigoni D. Nguyen C. et al. (2024). Transient nicotine exposure in early adolescent male mice freezes their dopamine circuits in an immature state. *Nat. Commun.* 15:9017. 10.1038/s41467-024-53327-w 39424848 PMC11489768

[B145] Rooy M. Lazarevich I. Koukouli F. Maskos U. Gutkin B. (2021). Cholinergic modulation of hierarchical inhibitory control over cortical resting state dynamics: Local circuit modeling of schizophrenia-related hypofrontality. *Curr. Res. Neurobiol.* 2:100018. 10.1016/j.crneur.2021.100018 34820636 PMC8591733

[B146] Rose K. N. Schwarzschild M. A. Gomperts S. N. (2024). Clearing the smoke: What protects smokers from Parkinson’s disease? *Mov. Disord.* 39 267–272. 10.1002/mds.29707 38226487 PMC10923097

[B147] Rybnicek J. Chen Y. Milic M. Tio E. S. McLaurin J. Hohman T. J. et al. (2024). *CHRNA5* links chandelier cells to severity of amyloid pathology in aging and Alzheimer’s disease. *Transl. Psychiatry* 14:83. 10.1038/s41398-024-02785-3 38331937 PMC10853183

[B148] Sabec M. H. Savage Q. R. Wood J. L. Maskos U. (2025). Targeting high-affinity nicotinic receptors protects against the functional consequences of β-amyloid in mouse hippocampus. *Mol. Psychiatry* 30 556–566. 10.1038/s41380-024-02666-7 39164528

[B149] Sawyer S. M. Azzopardi P. S. Wickremarathne D. Patton G. C. (2018). The age of adolescence. *Lancet Child Adolesc. Health* 2 223–228. 10.1016/S2352-4642(18)30022-1 30169257

[B150] Schizophrenia Working Group of the Psychiatric Genomics Consortium (2014). Biological insights from 108 schizophrenia-associated genetic loci. *Nature* 511 421–427. 10.1038/nature13595 25056061 PMC4112379

[B151] Schmaljohann J. Minnerop M. Karwath P. Gündisch D. Falkai P. Guhlke S. et al. (2004). Imaging of central nAChReceptors with 2-[18F]F-A85380: Optimized synthesis and in vitro evaluation in Alzheimer’s disease. *Appl. Radiat. Isotopes* 61 1235–1240. 10.1016/j.apradiso.2004.02.026 15388115

[B152] Scholze P. Huck S. (2020). The α5 nicotinic acetylcholine receptor subunit differentially modulates α4β2* and α3β4* receptors. *Front. Synaptic Neurosci.* 12:607959. 10.3389/fnsyn.2020.607959 33343327 PMC7744819

[B153] Sciaccaluga M. Moriconi C. Martinello K. Catalano M. Bermudez I. Stitzel J. A. et al. (2015). Crucial role of nicotinic α5 subunit variants for Ca2+ fluxes in ventral midbrain neurons. *FASEB J.* 29 3389–3398. 10.1096/fj.14-268102 25911614 PMC4511205

[B154] Sérrière S. Doméné A. Vercouillie J. Mothes C. Bodard S. Rodrigues N. et al. (2015). Assessment of the protection of dopaminergic neurons by an *A*7 nicotinic receptor agonist, PHA 543613 using [18F]LBT-999 in a Parkinson’s disease rat model. *Front. Med.* 2:61. 10.3389/fmed.2015.00061 26389120 PMC4556971

[B155] Shafiee N. Fonov V. Dadar M. Spreng R. N. Collins D. L. (2024). Degeneration in nucleus basalis of meynert signals earliest stage of Alzheimer’s disease progression. *Neurobiol. Aging* 139 54–63. 10.1016/j.neurobiolaging.2024.03.003 38608458

[B156] Shannon H. E. Rasmussen K. Bymaster F. P. Hart J. C. Peters S. C. Swedberg M. D. et al. (2000). Xanomeline, an M(1)/M(4) preferring muscarinic cholinergic receptor agonist, produces antipsychotic-like activity in rats and mice. *Schizophr. Res.* 42 249–259. 10.1016/s0920-9964(99)00138-3 10785583

[B157] Shim J. C. Jung D. U. Jung S. S. Seo Y. S. Cho D. M. Lee J. H. et al. (2012). Adjunctive varenicline treatment with antipsychotic medications for cognitive impairments in people with schizophrenia: A randomized double-blind placebo-controlled trial. *Neuropsychopharmacology* 37 660–668. 10.1038/npp.2011.238 22048460 PMC3260973

[B158] Shimohama S. Kawamata J. (2018). “Roles of Nicotinic Acetylcholine Receptors in the Pathology and Treatment of Alzheimer’s and Parkinson’s Diseases,” in *Nicotinic Acetylcholine Receptor Signaling in Neuroprotection*, eds Akaike A. Shimohama S. Misu Y. (Berlin: Springer). 31314416

[B159] Shlomo D. Zurita H. Valero M. Abad-Perez P. Kruglikov I. Meng J. et al. (2025). Inhibitory and disinhibitory VIP IN-Mediated circuits in neocortex. *bioRxiv [Preprint]* 10.1101/2025.02.26.640383 40060562 PMC11888407

[B160] Shram M. J. Funk D. Li Z. Lê A. D. (2007). Acute nicotine enhances c-fos mRNA expression differentially in reward-related substrates of adolescent and adult rat brain. *Neurosci. Lett.* 418 286–291. 10.1016/j.neulet.2007.03.034 17420096

[B161] Sinkus M. L. Graw S. Freedman R. Ross R. G. Lester H. A. Leonard S. (2015). The human CHRNA7 and CHRFAM7A genes: A review of the genetics, regulation, and function. *Neuropharmacology* 96 274–288. 10.1016/j.neuropharm.2015.02.006 25701707 PMC4486515

[B162] Smith R. C. Amiaz R. Si T. M. Maayan L. Jin H. Boules S. et al. (2016). Varenicline effects on smoking, cognition, and psychiatric symptoms in schizophrenia: A double-blind randomized trial. *PLoS One* 11:e0143490. 10.1371/journal.pone.0143490 26730716 PMC4701439

[B163] Smoller J. W. Andreassen O. A. Edenberg H. J. Faraone S. V. Glatt S. J. Kendler K. S. (2019). Psychiatric genetics and the structure of psychopathology. *Mol. Psychiatry* 24 409–420. 10.1038/s41380-017-0010-4 29317742 PMC6684352

[B164] Stefansson H. Rujescu D. Cichon S. Pietiläinen O. P. Ingason A. Steinberg S. et al. (2008). Large recurrent microdeletions associated with schizophrenia. *Nature* 455 232–236. 10.1038/nature07229 18668039 PMC2687075

[B165] Stoiljkovic M. Kelley C. Nagy D. Hurst R. Hajós M. (2016). Activation of α7 nicotinic acetylcholine receptors facilitates long-term potentiation at the hippocampal-prefrontal cortex synapses *in vivo*. *Eur. Neuropsychopharmacol.* 26 2018–2023. 10.1016/j.euroneuro.2016.11.003 27866776

[B166] Styr B. Slutsky I. (2018). Imbalance between firing homeostasis and synaptic plasticity drives early-phase Alzheimer’s disease. *Nat. Neurosci.* 21 463–473. 10.1038/s41593-018-0080-x 29403035 PMC6533171

[B167] Szigeti K. Ihnatovych I. Birkaya B. Chen Z. Ouf A. Indurthi D. C. et al. (2020). CHRFAM7A: A human specific fusion gene, accounts for the translational gap for cholinergic strategies in Alzheimer’s disease. *EBioMedicine* 59:102892. 10.1016/j.ebiom.2020.102892 32818803 PMC7452451

[B168] Takeshima T. Takeuchi H. Egawa T. Konaka S. (2007). Molecular structure of cotinine studied by gas electron diffraction combined with theoretical calculations. *J. Mol. Struct.* 841 13–21. 10.1016/j.molstruc.2006.11.041

[B169] Taly A. Corringer P. J. Guedin D. Lestage P. Changeux J. P. (2009). Nicotinic receptors: Allosteric transitions and therapeutic targets in the nervous system. *Nat. Rev. Drug Discov.* 8 733–750. 10.1038/nrd2927 19721446

[B170] Tapper A. R. McKinney S. L. Nashmi R. Schwarz J. Deshpande P. Labarca C. et al. (2004). Nicotine activation of alpha4* receptors: Sufficient for reward, tolerance, and sensitization. *Science* 306 1029–1032. 10.1126/science.1099420 15528443

[B171] Tasic B. Yao Z. Graybuck L. T. Smith K. A. Nguyen T. N. Bertagnolli D. et al. (2018). Shared and distinct transcriptomic cell types across neocortical areas. *Nature* 563 72–78. 10.1038/s41586-018-0654-5 30382198 PMC6456269

[B172] Thomas A. M. Ostroumov A. Kimmey B. A. Taormina M. B. Holden W. M. Kim K. et al. (2018). Adolescent nicotine exposure alters GABAA receptor signaling in the ventral tegmental area and increases adult ethanol self-administration. *Cell Rep.* 23 68–77. 10.1016/j.celrep.2018.03.030 29617674 PMC5983379

[B173] Titus D. J. Johnstone T. Johnson N. H. London S. H. Chapalamadugu M. Hogenkamp D. et al. (2019). Positive allosteric modulation of the α7 nicotinic acetylcholine receptor as a treatment for cognitive deficits after traumatic brain injury. *PLoS One* 14:e0223180. 10.1371/journal.pone.0223180 31581202 PMC6776323

[B174] Tobin A. B. (2024). A golden age of muscarinic acetylcholine receptor modulation in neurological diseases. *Nat. Rev. Drug Discov.* 23 743–758. 10.1038/s41573-024-01007-1 39143241

[B175] Tochitsky I. Banghart M. R. Mourot A. Yao J. Z. Gaub B. Kramer R. H. et al. (2012). Optochemical control of genetically engineered neuronal nicotinic acetylcholine receptors. *Nat. Chem.* 4 105–111. 10.1038/nchem.1234 22270644 PMC4977190

[B176] Townsend M. Whyment A. Walczak J. S. Jeggo R. van den Top M. Flood D. G. et al. (2016). α7-nAChR agonist enhances neural plasticity in the hippocampus via a GABAergic circuit. *J. Neurophysiol.* 116 2663–2675. 10.1152/jn.00243.2016 27655963 PMC5133305

[B177] Trubetskoy V. Pardiñas A. F. Qi T. Panagiotaropoulou G. Awasthi S. Bigdeli T. B. et al. (2022). Mapping genomic loci implicates genes and synaptic biology in schizophrenia. *Nature* 604 502–508. 10.1038/s41586-022-04434-5 35396580 PMC9392466

[B178] Turnbull M. T. Boskovic Z. Coulson E. J. (2018). Acute down-regulation of BDNF signaling does not replicate exacerbated Amyloid-β levels and cognitive impairment induced by cholinergic basal forebrain lesion. *Front. Mol. Neurosci.* 11:51. 10.3389/fnmol.2018.00051 29520217 PMC5827359

[B179] Udakis M. Wright V. L. Wonnacott S. Bailey C. P. (2016). Integration of inhibitory and excitatory effects of α7 nicotinic acetylcholine receptor activation in the prelimbic cortex regulates network activity and plasticity. *Neuropharmacology* 105 618–629. 10.1016/j.neuropharm.2016.02.028 26921769 PMC4881417

[B180] Ullah I. Zhao L. Uddin S. Zhou Y. Wang X. Li H. (2024). Nicotine-mediated therapy for Parkinson’s disease in transgenic Caenorhabditis elegans model. *Front. Aging Neurosci.* 16:1358141. 10.3389/fnagi.2024.1358141 38813528 PMC11135287

[B181] Venkatesan S. Lambe E. K. (2020). Chrna5 is essential for a rapid and protected response to optogenetic release of endogenous acetylcholine in prefrontal cortex. *J. Neurosci.* 40 7255–7268. 10.1523/JNEUROSCI.1128-20.2020 32817066 PMC7534913

[B182] Venkatesan S. Chen T. Liu Y. Turner E. E. Tripathy S. J. Lambe E. K. (2023). Chrna5 and lynx prototoxins identify acetylcholine super-responder subplate neurons. *iScience* 26:105992. 10.1016/j.isci.2023.105992 36798433 PMC9926215

[B183] Vico Varela E. Etter G. Williams S. (2019). Excitatory-inhibitory imbalance in Alzheimer’s disease and therapeutic significance. *Neurobiol. Dis.* 127 605–615. 10.1016/j.nbd.2019.04.010 30999010

[B184] Walling D. Marder S. R. Kane J. Fleischhacker W. W. Keefe R. S. Hosford D. A. et al. (2016). Phase 2 trial of an Alpha-7 nicotinic receptor agonist (TC-5619) in negative and cognitive symptoms of schizophrenia. *Schizophr. Bull.* 42 335–343. 10.1093/schbul/sbv072 26071208 PMC4753586

[B185] Wang H. Y. Lee D. H. D’Andrea M. R. Peterson P. A. Shank R. P. Reitz A. B. (2000). beta-Amyloid(1-42) binds to alpha7 nicotinic acetylcholine receptor with high affinity. implications for Alzheimer’s disease pathology. *J. Biol. Chem.* 275 5626–5632. 10.1074/jbc.275.8.5626 10681545

[B186] Wang X. L. Deng Y. X. Gao Y. M. Dong Y. T. Wang F. Guan Z. Z. et al. (2020). Activation of *A*7 nAChR by PNU-282987 improves synaptic and cognitive functions through restoring the expression of synaptic-associated proteins and the CaM-CaMKII-CREB signaling pathway. *Aging* 12 543–570. 10.18632/aging.102640 31905173 PMC6977648

[B187] White H. K. Levin E. D. (2004). Chronic transdermal nicotine patch treatment effects on cognitive performance in age-associated memory impairment. *Psychopharmacology* 171 465–471. 10.1007/s00213-003-1614-8 14534771

[B188] Whitehouse P. J. Price D. L. Clark A. W. Coyle J. T. DeLong M. R. (1981). Alzheimer disease: Evidence for selective loss of cholinergic neurons in the nucleus basalis. *Annals Neurol.* 10 122–126. 10.1002/ana.410100203 7283399

[B189] Wright V. L. Georgiou P. Bailey A. Heal D. J. Bailey C. P. Wonnacott S. (2019). Inhibition of Alpha7 nicotinic receptors in the ventral hippocampus selectively attenuates reinstatement of morphine-conditioned place preference and associated changes in AMPA receptor binding. *Addict. Biol.* 24 590–603. 10.1111/adb.12624 29667304 PMC6563460

[B190] Wu J. Ishikawa M. Zhang J. Hashimoto K. (2010). Brain imaging of nicotinic receptors in Alzheimer’s disease. *Intern. J. Alzheimer’s Dis.* 2010:548913. 10.4061/2010/548913 21253523 PMC3022172

[B191] Xu H. Garcia-Ptacek S. Jönsson L. Wimo A. Nordström P. Eriksdotter M. (2021). Long-Term effects of cholinesterase inhibitors on cognitive decline and mortality. *Neurology* 96 e2220–e2230. 10.1212/WNL.0000000000011832 33741639 PMC8166426

[B192] Yang D. Qi G. Delev D. Maskos U. Feldmeyer D. (2025). Linking altered neuronal and synaptic properties to nicotinic receptor Alpha5 subunit gene dysfunction: A translational investigation in rat mPFC and human cortical layer 6. *Transl. Psychiatry* 15:12. 10.1038/s41398-025-03230-9 39824806 PMC11748723

[B193] Yang J. Liu A. Y. Tang B. Luo D. Lai Y. J. Zhu B. L. et al. (2017). Chronic nicotine differentially affects murine transcriptome profiling in isolated cortical interneurons and pyramidal neurons. *BMC Genom.* 18:194. 10.1186/s12864-017-3593-x 28219337 PMC5319194

[B194] Young J. W. Finlayson K. Spratt C. Marston H. M. Crawford N. Kelly J. S. et al. (2004). Nicotine improves sustained attention in mice: Evidence for involvement of the *A*7 nicotinic acetylcholine receptor. *Neuropsychopharmacology* 29 891–900. 10.1038/sj.npp.1300393 14970827

[B195] Yuan M. Cross S. J. Loughlin S. E. Leslie F. M. (2015). Nicotine and the adolescent brain. *J. Physiol.* 593(Pt 16), 3397–3412. 10.1113/JP270492 26018031 PMC4560573

[B196] Zhu Y. Wu T. Jiao Q. Chai H. Wang Y. Tian C. et al. (2025). Acute REM sleep deprivation alleviated depression-like behavior mediated by inhibiting VIP Neurons in the mPFC. *Sci. Adv.* 11:eadx2666. 10.1126/sciadv.adx2666 40929273 PMC12422183

